# Pseudomonas aeruginosa Increases the Sensitivity of Biofilm-Grown Staphylococcus aureus to Membrane-Targeting Antiseptics and Antibiotics

**DOI:** 10.1128/mBio.01501-19

**Published:** 2019-07-30

**Authors:** Giulia Orazi, Kathryn L. Ruoff, George A. O’Toole

**Affiliations:** a Department of Microbiology and Immunology, Geisel School of Medicine at Dartmouth, Hanover, New Hampshire, USA; University of Rochester; McMaster University; Harvard Medical School

**Keywords:** *Pseudomonas aeruginosa*, *Staphylococcus aureus*, antibiotics, biofilm, membrane

## Abstract

Pseudomonas aeruginosa and Staphylococcus aureus often cause chronic, recalcitrant infections in large part due to their ability to form biofilms. The biofilm mode of growth enables these organisms to withstand antibacterial insults that would effectively eliminate their planktonic counterparts. We found that P. aeruginosa supernatant increased the sensitivity of S. aureus biofilms to multiple antimicrobial compounds, including fluoroquinolones and membrane-targeting antibacterial agents, including the antiseptic chloroxylenol. Treatment of S. aureus with the antiseptic chloroxylenol alone did not decrease biofilm cell viability; however, the combination of chloroxylenol and P. aeruginosa supernatant led to a 4-log reduction in S. aureus biofilm viability compared to exposure to chloroxylenol alone. We found that the P. aeruginosa-produced small molecule 2-*n*-heptyl-4-hydroxyquinoline *N*-oxide (HQNO) is responsible for the observed heightened sensitivity of S. aureus to chloroxylenol. Similarly, HQNO increased the susceptibility of S. aureus biofilms to other compounds, including both traditional and nontraditional antibiotics, which permeabilize bacterial membranes. Genetic and phenotypic studies support a model whereby HQNO causes an increase in S. aureus membrane fluidity, thereby improving the efficacy of membrane-targeting antiseptics and antibiotics. Importantly, our data show that P. aeruginosa exoproducts can enhance the ability of various antimicrobial agents to kill biofilm populations of S. aureus that are typically difficult to eradicate. Finally, our discovery that altering membrane fluidity shifts antimicrobial sensitivity profiles of bacterial biofilms may guide new approaches to target persistent infections, such as those commonly found in respiratory tract infections and in chronic wounds.

## Abstract (precis)

The thick mucus in the airways of cystic fibrosis (CF) patients predisposes them to frequent, polymicrobial respiratory infections. Pseudomonas aeruginosa and Staphylococcus aureus are frequently coisolated from the airways of individuals with CF, as well as from diabetic foot ulcers and other wounds. Both organisms form biofilms, which are notoriously difficult to eradicate and promote chronic infection. In this study, we have shown that P. aeruginosa-secreted factors can increase the efficacy of compounds that alone have little or no bactericidal activity against S. aureus biofilms. In particular, we discovered that P. aeruginosa exoproducts can potentiate the antistaphylococcal activity of phenol-based antiseptics and other membrane-active drugs. Our findings illustrate that polymicrobial interactions can dramatically increase antibacterial efficacy *in vitro* and suggest that altering membrane physiology promotes the ability of certain drugs to kill bacterial biofilms—knowledge that may provide a path for the discovery of new biofilm-targeting antimicrobial strategies.

## INTRODUCTION

Bacterial biofilms are the underlying cause of many chronic, difficult-to-treat infections. The biofilm lifestyle confers high-level tolerance to antibiotics and antiseptics, which is reflected by the requirement of 100- to 1,000-times-higher concentrations of these compounds to treat biofilms compared to their planktonic counterparts (1). As a result, it has proven difficult to find treatments that effectively eradicate biofilms (2–4).

Studies assessing biofilm antibiotic and antiseptic tolerance have typically been performed with single-species biofilms. While such single-species communities are commonly associated with implant infections (5), many infections are caused by polymicrobial biofilms, including respiratory infections, otitis media, urinary tract infections, and infections of both surgical and chronic wounds (6–19). Emerging evidence suggests that growth in these mixed microbial communities can alter antimicrobial tolerance profiles, often in unexpected ways (20–40), but the mechanism(s) underlying such altered tolerance is often poorly understood, with some exceptions. For example, a previous study from our group showed that secreted products of Pseudomonas aeruginosa could enhance biofilm tolerance of Staphylococcus aureus to vancomycin by 100-fold, likely via interfering with the function of the electron transport chain and slowing growth of S. aureus (37).

P. aeruginosa and S. aureus coexist in multiple infection settings, and both form biofilms that can be difficult to eradicate. P. aeruginosa and S. aureus are two of the most prevalent respiratory pathogens in patients with cystic fibrosis (CF) and are both associated with poor lung function and clinical outcomes in these patients (41–45). CF patients who are coinfected with P. aeruginosa and S. aureus have worse outcomes than those who are infected with either organism alone (46–50). In addition, P. aeruginosa and S. aureus are often coisolated from chronic wounds, including difficult-to-treat diabetic foot ulcers (51, 52). Furthermore, *in vitro* evidence suggests that P. aeruginosa and S. aureus coinfection delays wound healing (53).

In this study, we have identified several compounds that alone have little activity against S. aureus biofilms, but when combined with secreted products from P. aeruginosa, these agents can effectively decrease S. aureus biofilm viability. We propose a model whereby the P. aeruginosa exoproduct 2-*n*-heptyl-4-hydroxyquinoline *N*-oxide (HQNO) interacts with the S. aureus cell membrane, which leads to increased membrane fluidity and potentiates the ability of membrane-active compounds to more effectively target S. aureus biofilms.

## RESULTS

### P. aeruginosa supernatant increases S. aureus sensitivity to multiple antibiotic compounds.

In a previous study, we found that P. aeruginosa exoproducts decrease the efficacy of vancomycin against S. aureus biofilms (37). To test whether P. aeruginosa might impact S. aureus sensitivity to other antibiotics, we screened Biolog Phenotype MicroArray panels for changes in S. aureus antibiotic sensitivity in the presence versus absence of P. aeruginosa cell-free culture supernatant. Specifically, we tested MicroArray panels 11 to 20, which contain 240 antibacterial compounds. We identified many compounds that became either less effective, as reported previously (37), or, as we show here, more effective at killing S. aureus when in the presence of P. aeruginosa exoproducts (see Table S1 in the supplemental material). Increased efficacy of a drug was defined as at least a 10-fold decrease in CFU between S. aureus exposed to the antibiotic alone and S. aureus exposed to P. aeruginosa supernatant plus the antibiotic. Of the 240 compounds tested, 107 became more effective against S. aureus biofilm populations in the initial screen (Table S1).

10.1128/mBio.01501-19.8 TABLE S1 P. aeruginosa supernatant increases S. aureus biofilm sensitivity to antiseptics and antibiotics. Download Table S1, PDF file, 0.1 MB. Copyright © 2019 Orazi et al. 2019 Orazi et al. This content is distributed under the terms of the Creative Commons Attribution 4.0 International license.

Among the several classes of antimicrobial agents that became more effective at killing S. aureus in the presence of P. aeruginosa supernatant are nucleic acid synthesis inhibitors, membrane-active antibiotics, and antiseptics. Additionally, we identified other compounds not typically used to treat bacterial infections that became more effective at decreasing S. aureus viability, including anticholinergic agents, antipsychotic drugs, and ion channel blockers (Table S1).

### P. aeruginosa supernatant increases S. aureus biofilm sensitivity to chloroxylenol.

In the experiments described above using the Biolog Phenotype MicroArray panels, the compounds tested were added at the same time as the microbes were inoculated into the medium; thus, there was limited time for the bacteria to form a biofilm before exposure to the candidate agents. Therefore, we next tested whether P. aeruginosa supernatant could increase the efficacy of the compounds we identified in the Biolog screen against preformed early (6-h) S. aureus biofilms. In these experiments, the biofilm of S. aureus Newman was allowed to form for 6 h, and fresh medium supplemented with the indicated compound and/or P. aeruginosa supernatant was added to this preformed biofilm. This method is what we refer to as the biofilm disruption assay, described in more detail in the supplemental materials and methods (Text S1). Previously, we showed that by 6 h postinoculation (p.i.), the adherent population of S. aureus Newman cells is tolerant to vancomycin; at this time point, there is a difference of 3 logs between the cell viability of the biofilm population and that of the planktonic population for a given dose of antibiotic (37). Thus, these communities have one of the key phenotypic traits of a biofilm.

10.1128/mBio.01501-19.1 TEXT S1 Supplemental results and materials and methods. Download Text S1, PDF file, 0.2 MB. Copyright © 2019 Orazi et al. 2019 Orazi et al. This content is distributed under the terms of the Creative Commons Attribution 4.0 International license.

Of the 106 compounds that became more effective at killing S. aureus biofilms in the original screen, 42 compounds were tested against preformed early (6-h) S. aureus biofilms, which were representatives of a variety of drug classes (Table S1). Out of the 42 compounds tested, 6 became more effective at killing preformed S. aureus biofilms when in the presence of P. aeruginosa supernatant (Table S1).

**FIG 1:**
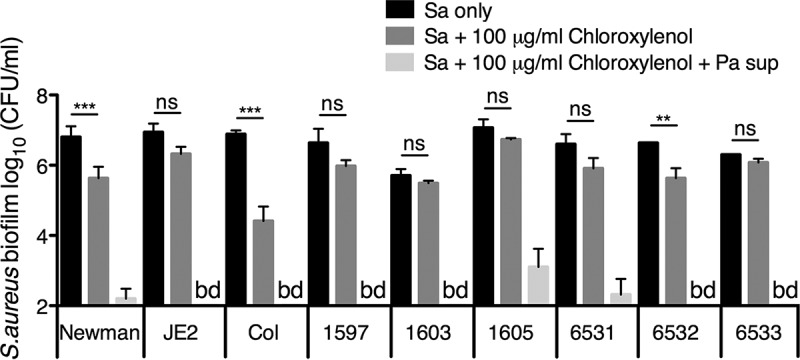
P. aeruginosa supernatant increases S. aureus biofilm sensitivity to chloroxylenol. Biofilm disruption assays on plastic were performed with the specified S. aureus clinical isolate, P. aeruginosa PA14 supernatant (Pa sup), and chloroxylenol at 100 μg/ml. Biofilms were grown for 6 h and exposed to the above treatments for 18 h, and S. aureus biofilm CFU were determined. Each column displays the average from two biological replicates, each with three technical replicates. Error bars indicate standard deviation (SD). Sa, S. aureus; bd, below detection; ns, not significant; **, *P* < 0.01; ***, *P* < 0.001, by ordinary one-way ANOVA and Bonferroni’s multiple-comparison posttest.

We found that P. aeruginosa supernatant increased the sensitivity of early (6-h) S. aureus biofilms to the topical antibiotic chloroxylenol (Fig. 1). Similarly to other phenol-based antiseptics, this compound impacts bacterial cell membranes, leading to increased fluidity and membrane permeability (54–56). Alone, chloroxylenol displayed modest activity against S. aureus biofilms. Strikingly, the ability of the antiseptic chloroxylenol to kill early S. aureus Newman biofilms was enhanced by 4 logs compared to the activity of chloroxylenol alone when combined with P. aeruginosa-secreted products (Fig. 1). We evaluated whether this phenotype is specific to the Newman strain or a more general phenomenon by testing multiple S. aureus laboratory strains and clinical isolates—both methicillin sensitive and methicillin resistant (Table S2). In all cases, we observed that P. aeruginosa supernatant dramatically increased the efficacy of chloroxylenol against S. aureus biofilms (Fig. 1). Chloroxylenol is dissolved in ethanol; we confirmed that the volume of ethanol used does not decrease S. aureus viability in either the presence or absence of P. aeruginosa supernatant (Fig. S1A). Moreover, the impact of supernatant on S. aureus sensitivity to chloroxylenol could be observed as early as 3 h after addition of the compounds to a 6-h-old biofilm, and the reduction in viability continued for 24 h posttreatment, wherein the assay was reaching its limit of detection (Fig. S1B).

10.1128/mBio.01501-19.2 FIG S1 The concentration of ethanol used in this study does not decrease S. aureus biofilm viability in the presence or absence of P. aeruginosa supernatant, and P. aeruginosa supernatant rapidly increases S. aureus biofilm sensitivity to chloroxylenol. Download FIG S1, PDF file, 0.2 MB. Copyright © 2019 Orazi et al. 2019 Orazi et al. This content is distributed under the terms of the Creative Commons Attribution 4.0 International license.

10.1128/mBio.01501-19.9 TABLE S2 Strains used in this study. Download Table S2, PDF file, 0.1 MB. Copyright © 2019 Orazi et al. 2019 Orazi et al. This content is distributed under the terms of the Creative Commons Attribution 4.0 International license.

### P. aeruginosa supernatant increases the ability of chloroxylenol to eradicate difficult-to-treat S. aureus biofilms.

**FIG 2:**
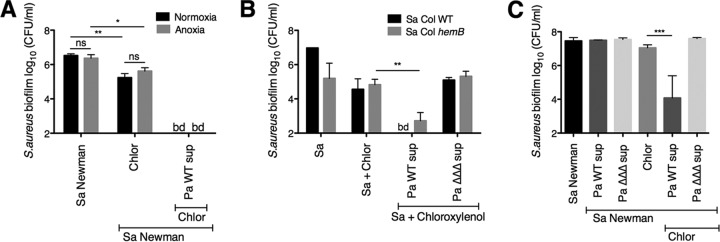
P. aeruginosa supernatant enhances the ability of chloroxylenol to kill difficult-to-treat S. aureus biofilms. (A) Biofilm disruption assays on plastic were performed with S. aureus (Sa) Newman, P. aeruginosa PA14 supernatant (Pa sup), and chloroxylenol (Chlor) at 100 μg/ml under normoxic or anoxic conditions. Biofilms were grown for 6 h and exposed to the above treatments for 18 h, and S. aureus biofilm CFU were determined. (B) Biofilm disruption assays on plastic were performed with S. aureus (Sa) Col parental strain or *hemB* mutant, supernatants from wild-type P. aeruginosa PA14 and the Δ*pqsL* Δ*pvdA* Δ*pchE* mutant (Pa ΔΔΔ sup), and chloroxylenol (Chlor) at 100 μg/ml. Biofilms were grown for 6 h and exposed to the above treatments for 18 h, and S. aureus biofilm CFU were determined. (C) Biofilm disruption assays on plastic were performed with S. aureus (Sa) Newman, supernatants from wild-type P. aeruginosa PA14 and the Δ*pqsL* Δ*pvdA* Δ*pchE* mutant (Pa ΔΔΔ sup), and chloroxylenol (Chlor) at 100 μg/ml. Biofilms were grown for 24 h and exposed to the above treatments for 24 additional hours, and S. aureus biofilm CFU were determined. Each column displays the average from three biological replicates, each with three technical replicates. Error bars indicate standard deviations. bd, below detection; ns, not significant; *, *P* < 0.05; **, *P* < 0.01; ***, *P* < 0.001, by ordinary one-way ANOVA and Tukey’s multiple-comparison posttest.

We then determined whether P. aeruginosa could enable chloroxylenol to kill especially difficult-to-treat S. aureus biofilms. S. aureus grown in anoxia and respiration-deficient S. aureus small colony variants (SCVs) both exhibit high tolerance to many classes of antibiotics (57–59), likely because the bacteria need to be actively growing in order for many antibacterial compounds to be effective. Depending on the antibiotic class, either the antibiotic target needs to be produced or electron transport is required for drug uptake (57, 60), but membrane-targeting agents are an exception; the target is present whether or not the organism is actively growing (61). Indeed, P. aeruginosa supernatant increased the efficacy of chloroxylenol against S. aureus Newman biofilms to similar degrees in anoxia and normoxia (Fig. 2A).

To test whether the combination of P. aeruginosa supernatant and chloroxylenol is effective against biofilm-grown S. aureus SCVs, we used an S. aureus Col strain that has a mutation in *hemB*, a gene involved in hemin biosynthesis. The S. aureus *hemB* mutant is defective in electron transport and has the typical characteristics of clinical SCVs (62). We observed that P. aeruginosa supernatant enhanced chloroxylenol’s activity against the Col *hemB* mutant as well as the parental strain (Fig. 2B).

Furthermore, we tested whether more mature S. aureus biofilms could be effectively targeted by the P. aeruginosa supernatant-chloroxylenol combination. When we grew S. aureus Newman biofilms for 24 h before exposure to the combination treatment, we observed a striking 4-log-fold enhancement of chloroxylenol’s antimicrobial activity (Fig. 2C), similar to what was seen for 6-h-grown biofilms (Fig. 1).

### The P. aeruginosa exoproducts HQNO and siderophores increase S. aureus biofilm and planktonic sensitivity to chloroxylenol.

To explore the mechanism underlying P. aeruginosa supernatant-mediated enhancement of chloroxylenol’s antistaphylococcal activity, we sought to identify P. aeruginosa mutants that were unable to increase the sensitivity of S. aureus Newman biofilms to this drug. Previously, we showed that 2-*n*-heptyl-4-hydroxyquinoline *N*-oxide (HQNO) and siderophores contribute to the ability of P. aeruginosa to protect S. aureus from vancomycin (37). Thus, we tested P. aeruginosa PA14 strains with mutations in genes encoding components of the *Pseudomonas* quinolone signal (PQS) quorum sensing system (*pqsA*, *pqsH*, and *pqsL*) and biosynthesis of the siderophores pyoverdine (*pvdA*) and pyochelin (*pchE*). Supernatants from P. aeruginosa PA14 Δ*pqsA*, Δ*pqsH*, Δ*pqsL*, and Δ*pvdA* Δ*pchE* mutants each had a defect in the ability to increase S. aureus Newman biofilm sensitivity to chloroxylenol relative to the wild-type P. aeruginosa PA14 (Fig. S2A and B).

10.1128/mBio.01501-19.3 FIG S2 Testing the ability of P. aeruginosa PA14 mutants defective in exoproduct production to increase S. aureus biofilm sensitivity to chloroxylenol. Download FIG S2, PDF file, 0.1 MB. Copyright © 2019 Orazi et al. 2019 Orazi et al. This content is distributed under the terms of the Creative Commons Attribution 4.0 International license.

Additionally, we tested P. aeruginosa PA14 strains with mutations in genes encoding the following secreted products: hydrogen cyanide (*hcnA* and *hcnB*), LasA protease (*lasA*), elastase (*lasB*), and rhamnolipids (*rhlA*). Supernatants from these mutants retained the ability to increase the sensitivity of S. aureus biofilms to chloroxylenol (Fig. S2A and B).

**FIG 3:**
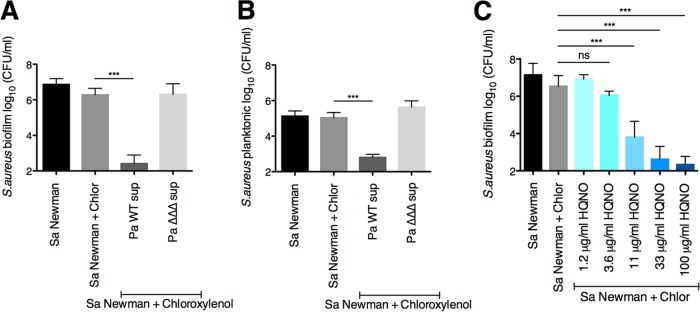
The P. aeruginosa exoproducts HQNO and siderophores increase S. aureus biofilm and planktonic sensitivity to chloroxylenol. (A and B) Biofilm disruption assays on plastic were performed with S. aureus (Sa) Newman, supernatants from wild-type P. aeruginosa PA14 and the Δ*pqsL* Δ*pvdA* Δ*pchE* deletion mutant (Pa ΔΔΔ sup), and chloroxylenol (Chlor) at 100 μg/ml. Biofilms were grown for 6 h and exposed to the above treatments for 18 h, and S. aureus biofilm (A) and planktonic (B) CFU were determined. Data in panels A and B were from the same experiments. (C) Biofilm disruption assays on plastic were performed with S. aureus (Sa) Newman, chloroxylenol (Chlor) at 100 μg/ml, and the specified concentrations of HQNO (dissolved in DMSO). Biofilms were grown for 6 h and exposed to the above treatments for 18 h, and S. aureus biofilm CFU were determined. Each column displays the average from at least three biological replicates, each with three technical replicates. Error bars indicate SD. ns, not significant; ***, *P* < 0.001, by ordinary one-way ANOVA and Tukey’s multiple-comparison posttest.

To investigate whether HQNO, pyoverdine, and pyochelin all contributed to the phenotype, we tested whether the supernatant from P. aeruginosa strains with mutations in the genes encoding all three factors was deficient in enhancing chloroxylenol’s activity against S. aureus. Indeed, supernatant from the P. aeruginosa PA14 Δ*pqsL* Δ*pvdA* Δ*pchE* mutant (designated the ΔΔΔ mutant) was unable to increase the sensitivity of S. aureus Newman biofilms to chloroxylenol (Fig. 3A and Fig. S2B). Supernatant from the P. aeruginosa PA14 Δ*pqsL* Δ*pvdA* Δ*pchE* mutant was unable to potentiate the ability of chloroxylenol to kill difficult-to-treat SCVs and 24-h-grown biofilms (Fig. 2B and C; Pa ΔΔΔ sup). Similarly to the biofilm population, we observed that P. aeruginosa PA14 wild-type supernatant, but not the Δ*pqsL* Δ*pvdA* Δ*pchE* mutant, enhances the ability of chloroxylenol to kill planktonic S. aureus Newman by approximately 3 logs (Fig. 3B). Thus, our data indicate that HQNO and both siderophores are required for P. aeruginosa-mediated enhancement of chloroxylenol’s activity against both planktonic and biofilm populations of S. aureus.

### HQNO alone enhances the activity of chloroxylenol against S. aureus biofilms.

To test whether HQNO alone could enhance to the ability of chloroxylenol to kill S. aureus in biofilm, we performed a biofilm disruption assay using commercially available HQNO. We used concentrations of HQNO that are in the range of those produced by P. aeruginosa PA14 under our experimental conditions (37), as well as those produced by stationary-phase P. aeruginosa cultures grown in rich medium (63, 64). Previously, we quantified the level of HQNO produced by P. aeruginosa PA14 after 24 h of growth in minimal medium on plastic plates, which is the source of P. aeruginosa supernatants used throughout this study (37). We found that the level of HQNO in these P. aeruginosa supernatants is ∼10 μg/ml. Additionally, P. aeruginosa PA14 produced ∼15 μg/ml HQNO when grown on CF-derived epithelial cells for 6 h (37). We observed a dose-response whereby increasing concentrations of exogenous HQNO corresponded with enhanced ability of chloroxylenol to kill S. aureus Newman biofilms (Fig. 3C). These results indicate that the presence of a single secreted factor, HQNO, is sufficient to alter S. aureus biofilm sensitivity to chloroxylenol.

### HQNO likely does not increase S. aureus sensitivity to chloroxylenol via inhibition of the ETC.

HQNO is well known to inhibit electron transport chain (ETC) complexes II and III in both mammalian and bacterial cells (65–68). To investigate whether HQNO shifts S. aureus sensitivity to chloroxylenol by inhibiting respiration, we tested the following ETC inhibitors: 3-nitropropionic acid (3-NP; complex II inhibitor), antimycin A (complex III inhibitor), sodium azide (azide; complex IV inhibitor), and oligomycin (ATP synthase inhibitor) or mutations in components of ATP synthase. All but one of the compounds tested, antimycin A, had little to no impact on S. aureus sensitivity to chloroxylenol, nor did mutations in the ATPase (Fig. S3A to E).

10.1128/mBio.01501-19.4 FIG S3 Testing the ability of electron transport chain inhibitors and a proton ionophore to increase S. aureus biofilm sensitivity to chloroxylenol. Download FIG S3, PDF file, 0.4 MB. Copyright © 2019 Orazi et al. 2019 Orazi et al. This content is distributed under the terms of the Creative Commons Attribution 4.0 International license.

It is possible that HQNO and antimycin A are changing antibiotic sensitivity not by inhibiting the ETC but via a different mechanism entirely. Thus, we took a different approach to investigate whether ETC inhibition changes S. aureus susceptibility to chloroxylenol. Exposure to anoxic conditions is a way to inhibit respiration that does not require the use of chemical compounds. Anoxia did not enhance chloroxylenol’s efficacy against S. aureus Newman biofilms in the absence of P. aeruginosa supernatant (Fig. 2A). Also, despite lacking a functional ETC, S. aureus SCVs are not hypersensitive to chloroxylenol (Fig. 2B). Furthermore, as we observed above, P. aeruginosa supernatant is able to potentiate the activity of chloroxylenol to kill SCVs even though these cells are respiration deficient (Fig. 2B). Together, these data indicate that HQNO likely alters S. aureus antibiotic sensitivity via a mechanism independent of its effects on the ETC.

We next considered several possible mechanisms underlying HQNO-mediated enhancement of chloroxylenol’s antistaphylococcal activity. Specifically, we tested the following models: (i) HQNO-mediated changes in membrane potential increase antibiotic sensitivity, (ii) HQNO-induced generation of reactive oxygen species leads to enhanced bacterial killing, (iii) HQNO alters the ability of S. aureus to efflux chloroxylenol, and/or (iv) HQNO changes properties of the S. aureus cell membrane. Experiments testing the first three of these models, which did not support these models, are presented in the supplemental results (Text S1) and in Fig. S3 and S4.

10.1128/mBio.01501-19.5 FIG S4 Reactive oxygen species do not sensitize S. aureus biofilms to chloroxylenol. Download FIG S4, PDF file, 0.3 MB. Copyright © 2019 Orazi et al. 2019 Orazi et al. This content is distributed under the terms of the Creative Commons Attribution 4.0 International license.

### Exogenous HQNO increases S. aureus membrane fluidity.

Previous studies have found that changes in the cell membrane fatty acid composition, which influences membrane fluidity, alter the susceptibility of bacterial cells to phenolic compounds (69). Thus, we tested whether HQNO might cause heightened susceptibility to chloroxylenol by altering the fluidity of the S. aureus cell membrane. To measure membrane fluidity, we performed Laurdan generalized polarization (GP) assays. Laurdan is a fluorescent dye that is sensitive to changes in membrane fluidity; the emission spectrum changes depending on the physical state of lipids within a bilayer. A decrease in Laurdan GP values corresponds to an increase in membrane fluidity. This dye has been previously used to measure the cell membrane fluidity of S. aureus (70–72).

**FIG 4:**
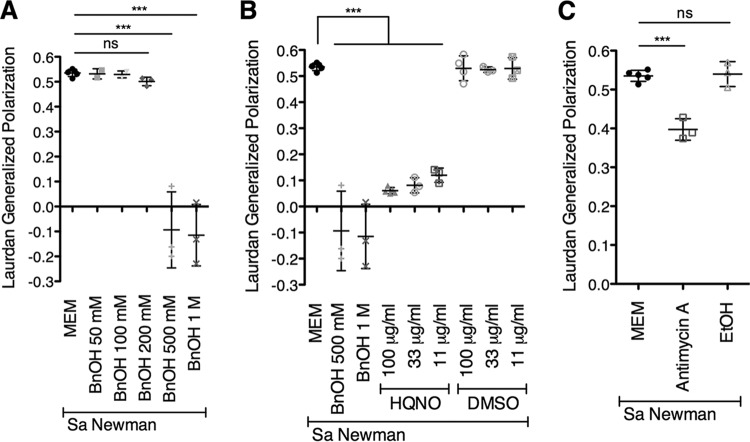
Exogenous HQNO increases S. aureus membrane fluidity. (A to C) Laurdan generalized polarization (GP) was performed with S. aureus (Sa) Newman, benzyl alcohol (BnOH) (A and B), HQNO (B), and the DMSO control (solvent for HQNO) (B) at the indicated concentrations and antimycin A at 100 μg/ml along with the ethanol (EtOH) control (solvent for antimycin A) (C). S. aureus was exposed to the above treatments for 1 h, and GP values were determined. Each column displays the average from at least three biological replicates, each with three technical replicates. Error bars indicate SD. ns, not significant; ***, *P* < 0.001, by ordinary one-way ANOVA and Tukey’s multiple-comparison posttest.

We used benzyl alcohol, a well-established membrane fluidizing agent (73–75), as a positive control. Exposure to 500 mM or 1 M benzyl alcohol for 1 h led to a significant decrease in Laurdan GP relative to S. aureus exposed to minimum essential medium (MEM), indicating an increase in membrane fluidity (Fig. 4A). We observed that treatment of S. aureus Newman with HQNO at all concentrations tested led to a significant reduction in Laurdan GP relative to exposure to MEM alone, indicating that HQNO has a fluidizing effect on the S. aureus membrane (Fig. 4B). Additionally, we found that exposure to antimycin A also led to a significant increase in fluidity (Fig. 4C), albeit to a lesser extent than HQNO (Fig. 4B). Furthermore, we showed that the solvents for HQNO and antimycin A, dimethyl sulfoxide (DMSO) and ethanol, respectively, did not cause the observed increase in S. aureus membrane fluidity (Fig. 4B and C).

### Shifting membrane fluidity alters S. aureus biofilm sensitivity to chloroxylenol.

**FIG 5:**
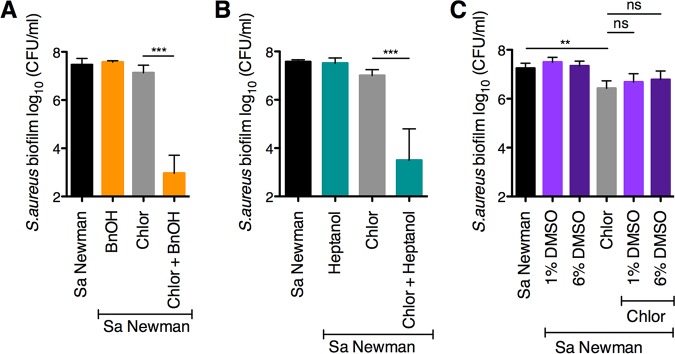
Shifting membrane fluidity alters S. aureus biofilm sensitivity to chloroxylenol. (A to C) Biofilm disruption assays on plastic were performed with S. aureus (Sa) Newman, chloroxylenol (Chlor) at 100 μg/ml, benzyl alcohol (BnOH) at 50 mM (A), 1-heptanol at 50 mM (B), and dimethyl sulfoxide (DMSO) at 1% and 6% (C). Biofilms were grown for 6 h and exposed to the above treatments for 18 h, and S. aureus biofilm CFU were determined. Each column displays the average from at least three biological replicates, each with three technical replicates. Error bars indicate SD. ns, not significant; **, *P* < 0.01; ***, *P* < 0.001, by ordinary one-way ANOVA and Tukey’s multiple-comparison posttest.

Next, we investigated whether the observed HQNO-mediated increase in membrane fluidity can lead to increased sensitivity to chloroxylenol. To test this hypothesis, we exposed S. aureus biofilms to various compounds that are known to influence membrane fluidity. Benzyl alcohol and 1-heptanol both impart higher fluidity, whereas dimethyl sulfoxide (DMSO) causes membranes to become less fluid (73–77). We observed that benzyl alcohol and 1-heptanol both increased S. aureus Newman biofilm sensitivity to chloroxylenol (Fig. 5A and B). In contrast, the membrane-rigidifying agent DMSO did not increase S. aureus Newman biofilm sensitivity to chloroxylenol (Fig. 5C). These results suggest that alterations in S. aureus membrane fluidity impact sensitivity to chloroxylenol, whereby increased fluidity leads to higher sensitivity.

Next, we showed that manipulating S. aureus fatty acid composition either by adding exogenous unsaturated fatty acids (Fig. S5A and Text S1) or by increasing the proportion of branched-chain fatty acids (BCFAs) relative to short-chain fatty acids (SCFAs) by mutation (Fig. S5B and Text S1) leads to increased S. aureus sensitivity to chloroxylenol. Additionally, we showed that decreasing levels of BCFAs relative to SCFAs by introducing the *lpd* mutation does not increase sensitivity to chloroxylenol (Fig. S5C) and that cardiolipin is not required for altered S. aureus sensitivity to this drug (Fig. S5D and Text S1).

10.1128/mBio.01501-19.6 FIG S5 Manipulating membrane fatty acid composition alters S. aureus biofilm sensitivity to chloroxylenol. Download FIG S5, PDF file, 0.3 MB. Copyright © 2019 Orazi et al. 2019 Orazi et al. This content is distributed under the terms of the Creative Commons Attribution 4.0 International license.

Together, our data suggest that changes in membrane fatty acid composition influence the efficacy of chloroxylenol and are consistent with our model that an increase in membrane fluidity promotes chloroxylenol’s ability to kill S. aureus biofilms.

### Prolonged exposure to P. aeruginosa exoproducts alters S. aureus membrane fatty acid profiles.

Our data above suggest that HQNO increases S. aureus membrane fluidity, which leads to heightened sensitivity of S. aureus to chloroxylenol. Thus, we explored whether HQNO induces changes in S. aureus membrane fatty acid composition. We performed a time course to track S. aureus fatty acid composition over time in the presence of P. aeruginosa exoproducts. Briefly, S. aureus Newman cells were exposed to medium alone (MEM + l-Gln) or P. aeruginosa PA14 wild-type supernatant for differing lengths of time (30 min, 1 h, 3 h, 6 h, or 10 h). Subsequently, fatty acid methyl ester (FAME) analysis was performed to measure the membrane fatty acid composition.

By 30 min or 1 h, the membrane fatty acid profile of S. aureus cells grown in medium alone appeared similar to the profile of P. aeruginosa supernatant-exposed S. aureus cells (Fig. S6A to C and Table S3). However, prolonged treatment with P. aeruginosa supernatant led to a shift in S. aureus membrane fatty acid profiles. In particular, S. aureus cells incubated with P. aeruginosa exoproducts for 24 h had significantly reduced relative BCFA levels compared to S. aureus grown in medium alone (Fig. S6D and E and Text S1). Above, we found that HQNO significantly increases S. aureus membrane fluidity after 1 h (Fig. 4B). Because the fluidizing effect of HQNO occurs more rapidly than the effect of P. aeruginosa supernatant on S. aureus membrane fatty acid composition, it is likely that the HQNO-mediated increase in S. aureus membrane fluidity that we observe does not occur via changes in membrane fatty acid profiles.

10.1128/mBio.01501-19.7 FIG S6 Exposure to P. aeruginosa exoproducts alters S. aureus membrane fatty acid composition. Download FIG S6, PDF file, 0.3 MB. Copyright © 2019 Orazi et al. 2019 Orazi et al. This content is distributed under the terms of the Creative Commons Attribution 4.0 International license.

10.1128/mBio.01501-19.10 TABLE S3 HQNO alters S. aureus membrane fatty acid profiles. Download Table S3, XLSX file, 0.02 MB. Copyright © 2019 Orazi et al. 2019 Orazi et al. This content is distributed under the terms of the Creative Commons Attribution 4.0 International license.

### P. aeruginosa supernatant increases S. aureus biofilm sensitivity to multiple membrane-targeting compounds.

**FIG 6:**
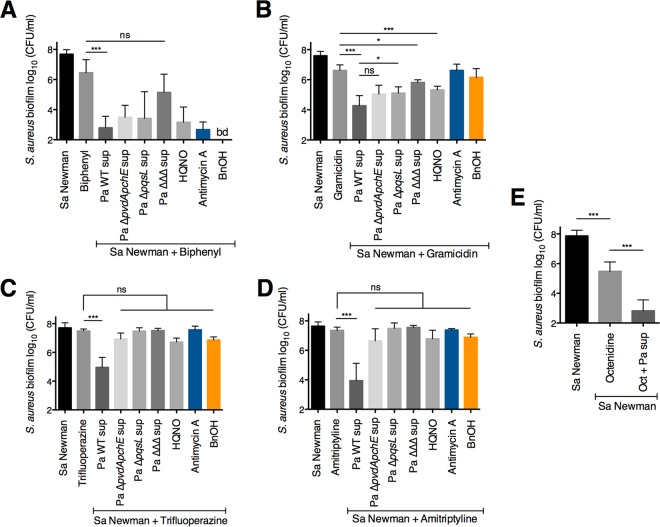
P. aeruginosa supernatant increases S. aureus biofilm sensitivity to other membrane-targeting compounds. (A to E) Biofilm disruption assays on plastic were performed with S. aureus (Sa) Newman; supernatants from wild-type P. aeruginosa PA14 and the specified mutants (Pa sup); and either biphenyl at 200 μg/ml (A), gramicidin at 100 μg/ml (B), trifluoperazine at 100 μg/ml (C), amitriptyline at 100 μg/ml (D), or octenidine dihydrochloride (Oct) at 5 μg/ml (E). Biofilms were grown for 6 h and exposed to the above treatments for 18 h, and S. aureus biofilm CFU were determined. Each column displays the average from at least three biological replicates, each with three technical replicates. Error bars indicate standard deviation (SD). ns, not significant; *, *P* < 0.05; ***, *P* < 0.001, by ordinary one-way ANOVA and Tukey’s multiple-comparison posttest.

Given the effects of P. aeruginosa exoproducts on S. aureus sensitivity to chloroxylenol, we explored whether P. aeruginosa alters the antistaphylococcal efficacy of other membrane-active antibiotics. Here, we tested the efficacy of the phenol-based antiseptic biphenyl, as well as the topical peptide antibiotic gramicidin in combination with P. aeruginosa supernatant. Both of these compounds are thought to kill bacteria by ultimately causing an increase in cell membrane permeability. We discovered that P. aeruginosa*-*secreted products enhance the ability of the membrane-active drugs biphenyl and gramicidin to kill S. aureus Newman biofilms (Fig. 6A and B). We also made the interesting observation that P. aeruginosa supernatant increases S. aureus biofilm sensitivity to two nontraditional antibiotics, trifluoperazine, an antipsychotic, and amitriptyline, an antidepressant (Fig. 6C and D). Strikingly, the combination of either of these drugs and P. aeruginosa supernatant led to a 2.5- to 3-log reduction in S. aureus biofilm viability compared to exposure to the drug alone (Fig. 6C and D). Supernatants from P. aeruginosa PA14 Δ*pqsL* and Δ*pvdA* Δ*pchE* mutants each had defects in the ability to increase S. aureus Newman biofilm sensitivity to trifluoperazine and amitriptyline relative to the wild-type P. aeruginosa PA14 (Fig. 6C and D), suggesting that HQNO and siderophores both contribute to this phenotype. In contrast, it appears than another P. aeruginosa-produced factor is involved in enhancing the activity of gramicidin against S. aureus biofilms (Fig. 6B).

Additionally, we examined whether altering membrane fluidity influenced S. aureus biofilm sensitivity to the above compounds. We observed that benzyl alcohol did not appreciably alter S. aureus sensitivity to gramicidin, trifluoperazine, or amitriptyline (Fig. 6B to D). In contrast, the fluidizing agent led to a striking increase in the antibacterial efficacy of biphenyl; the combination of these compounds led to a decrease in S. aureus Newman biofilm viability to below the level of detection of this assay (∼200 CFU/ml [Fig. 6A]). These results suggest that a more fluid membrane increases the susceptibility of S. aureus biofilms to biphenyl, which is a compound similar to chloroxylenol in structure and function.

Finally, we tested whether P. aeruginosa secreted products could increase the antistaphylococcal efficacy of octenidine dihydrochloride, a surfactant-based antiseptic that is approved for treatment of wound infections and has low cytotoxicity (78, 79). We observed that P. aeruginosa supernatant potentiates the activity of octenidine against S. aureus biofilms by 2.5 logs (Fig. 6E).

## DISCUSSION

In this study, we found that the interactions between two bacterial pathogens that are frequently coisolated from infections can cause striking and unexpected changes in antimicrobial susceptibility profiles. We showed that P. aeruginosa potentiates the ability of various antibacterial agents to kill S. aureus biofilms, which are often difficult to eradicate. In particular, we found that P. aeruginosa-secreted products increase the sensitivity of S. aureus biofilms to the topical antiseptic chloroxylenol. Alone, chloroxylenol at a concentration of 100 μg/ml is not effective at eradicating S. aureus biofilms; however, in combination with P. aeruginosa cell-free culture supernatant, which alone does not impact S. aureus viability, the efficacy of chloroxylenol increased 4-log-fold. Moreover, we have shown that P. aeruginosa supernatant can increase the ability of chloroxylenol to kill multiple strains and clinical isolates of S. aureus. Furthermore, we found that the small molecule HQNO and the siderophores pyoverdine and pyochelin contribute to the P. aeruginosa-mediated increase in the efficacy of chloroxylenol against S. aureus biofilms. In addition, we showed that HQNO alone recapitulated the effect of P. aeruginosa supernatant. Thus, the addition of a small molecule alone can greatly influence the efficacy of this antiseptic.

Previous studies have detected HQNO in expectorated sputum from CF patients infected with P. aeruginosa, and these levels are highly variable (29, 80). P. aeruginosa isolates from chronic CF pulmonary infections frequently have loss-of-function mutations in the quorum sensing regulator *lasR* and often overproduce alginate (81, 82). LasR inactivity and mucoidy each can lead to decreased HQNO production *in vitro* (64, 83). Therefore, quorum sensing activity and mucoidy may modulate the levels of HQNO produced by P. aeruginosa during infection and, in turn, influence the ability of HQNO to modify S. aureus drug sensitivity profiles *in vivo*.

HQNO has been shown to inhibit the S. aureus electron transport chain (ETC) (65). To investigate whether HQNO influences S. aureus susceptibility to chloroxylenol via inhibition of respiration, we treated S. aureus with chemical inhibitors of the ETC alone or in combination with the antibiotic. We found that only a subset of the ETC inhibitors tested increased the efficacy of chloroxylenol. However, anoxia did not increase S. aureus chloroxylenol sensitivity in the absence of HQNO. Additionally, despite having a defective ETC, S. aureus SCVs became more susceptible to chloroxylenol in the presence of HQNO, suggesting that inhibition of respiration is not required for this phenotype.

Since it is known that changes in membrane lipid profiles impact sensitivity to membrane-targeting compounds (69), we hypothesized that HQNO might cause heightened susceptibility to chloroxylenol by altering one or more properties of the S. aureus cell membrane. Like other phenol-based antiseptics, chloroxylenol is thought to insert into the cell membrane and cause an increase in membrane fluidity and permeability (54–56). Thus, an increase in membrane fluidity mediated by HQNO may allow for greater accumulation of chloroxylenol within the membrane and subsequently cause an increase in efficacy of the antibiotic. Manipulating the fluidity of E. coli membranes has been previously demonstrated to alter sensitivity to phenols, whereby decreasing membrane fluidity conferred increased tolerance to these compounds (69). Therefore, we tested whether HQNO changes the fluidity of the S. aureus cell membrane, potentially explaining the increased antimicrobial sensitivity we observe. We found that exogenous HQNO causes a striking increase in S. aureus membrane fluidity. Due to its hydrophobic character, it is plausible that HQNO directly interacts with the membrane to increase fluidity. In light of this result, we hypothesized that antimycin A and oligomycin, both hydrophobic compounds, also increase S. aureus sensitivity to chloroxylenol by altering membrane fluidity; the other ETC inhibitors tested, 3-NP and sodium azide, which did not enhance sensitivity to chloroxylenol, are both hydrophilic compounds. We showed that treatment of S. aureus with antimycin A also leads to an increase in membrane fluidity. These findings suggest that the observed HQNO-mediated increase in antibiotic efficacy is independent of the effect of HQNO on the S. aureus ETC. Furthermore, we showed that modulating membrane fluidity via either genetic or chemical approaches shifts S. aureus chloroxylenol sensitivity profiles. Together, these results are consistent with a model whereby HQNO increases S. aureus membrane fluidity, which greatly enhances the ability of chloroxylenol to kill S. aureus biofilms.

We also found that treatment with P. aeruginosa supernatant or pure HQNO influenced the membrane fatty acid composition of S. aureus. Specifically, S. aureus grown in medium alone had a significantly higher proportion of BCFA than did S. aureus cells exposed to P. aeruginosa supernatant or HQNO for 24 h. Given these results, we hypothesize that HQNO-mediated inhibition of the S. aureus ETC leads to decreased rates of fatty acid synthesis. Previous work from our laboratory has shown that when these organisms are in coculture, P. aeruginosa forces S. aureus to grow by fermentation (84), which leads to a reduction in growth of S. aureus (37). Furthermore, during coculture with P. aeruginosa, S. aureus downregulates multiple genes involved in fatty acid synthesis, including the cardiolipin synthase (*cls1*) and branched-chain amino acid transporters (*brnQ1*, *brnQ2*, *brnQ3*, and *bcaP*) (84). Additionally, it has been shown that anaerobically grown S. aureus has lower protein synthesis rates for multiple enzymes involved in metabolism, including FabG1, which is required for fatty acid synthesis (85).

Together, our results are consistent with the following two models, which are not mutually exclusive: (i) HQNO increases S. aureus membrane fluidity, potentially via direct interaction with the membrane, and (ii) exposure to HQNO slows or halts S. aureus fatty acid synthesis, leading to altered membrane lipid composition, perhaps via ETC inhibition. Our data suggest that the first model may explain how HQNO potentiates the activity of chloroxylenol against S. aureus biofilms. In contrast, our data do not support a role for the second model in explaining the altered chloroxylenol susceptibility profiles we observe. Specifically, the HQNO-mediated increase in S. aureus membrane fluidity occurs more rapidly than the P. aeruginosa supernatant-induced changes in fatty acid profiles. Therefore, we hypothesize that HQNO increases fluidity via direct interaction with the membrane, rather than via inducing a shift in membrane fatty acid composition. The second model could explain other potential consequences of this interspecies interaction, such as an impaired ability to adapt to changing environmental conditions.

We observed that P. aeruginosa exoproducts can potentiate the activity of multiple membrane-active compounds, including the phenol biphenyl and gramicidin, which forms channels within the membrane (86–88). Interestingly, we also showed that P. aeruginosa-secreted factors enhanced the activity of two nontraditional antibiotics, trifluoperazine and amitriptyline. Both of these drugs have a fused tricyclic structure and have been found to possess antibacterial activity (89–93). Additionally, trifluoperazine was found to synergize with fluconazole against multiple fungal species (94). Due to its high degree of hydrophobicity, trifluoperazine has been shown to interact with cell membranes and cause increased fluidity and permeability (95, 96); it has been hypothesized that amitriptyline acts in a similar manner (93).

Importantly, we found that the combination of P. aeruginosa supernatant and chloroxylenol was effective against multiple slow-growing S. aureus populations, namely, anaerobically grown biofilms and SCVs. Infection sites can have steep oxygen gradients (97, 98), which may lead to slow microbial growth *in vivo* (99). Slow-growing pathogens are difficult to eradicate because many antibiotic classes are effective against only actively growing cells; in contrast, antibacterial agents that target membranes are effective whether or not bacteria are growing. Thus, our discovery that an interspecies interaction can potentiate the activity of membrane-active drugs could be used to inform the treatment of recalcitrant mixed-species infections involving bacterial biofilms in oxygen-depleted sites.

Overall, our work demonstrates that polymicrobial interactions can profoundly shift the antibiotic sensitivity profiles of bacteria growing as biofilms. Furthermore, we discovered that interspecies interactions can lead to changes in the fluidity and composition of the bacterial cell membrane, which may influence other aspects of bacterial physiology as well as responses to environmental stressors. Additionally, our results suggest that manipulating membrane fluidity can influence the efficacy of various membrane-targeting drugs against bacterial biofilms. We propose that these findings could inspire new strategies for eradicating recalcitrant infections.

Because of its ability to inhibit mitochondrial respiration, HQNO is not a good candidate for a therapeutic; however, it is possible that other membrane-altering compounds could be used as adjuvants to antibacterial therapy. Together, our findings may have important consequences for the treatment of polymicrobial infections in multiple disease contexts, including nonhealing wounds and pulmonary infections in patients with cystic fibrosis.

## MATERIALS AND METHODS

See the supplemental materials and methods in Text S1 in the supplemental material for additional details regarding the methods.

### Bacterial strains and culture conditions.

A list of all strains used in this study is included in Table S2. S. aureus was grown in tryptic soy broth (TSB), and P. aeruginosa was grown in lysogeny broth (LB). All overnight cultures were grown with shaking at 37°C for 12 to 14 h, except for the S. aureus Col *hemB* mutant, which was grown statically at 37°C for 20 h.

### Biolog MicroArray antibiotic susceptibility assay.

Biolog Phenotype MicroArray bacterial chemical sensitivity assay panels were used to test S. aureus antimicrobial sensitivities as previously described (37). See the supplemental materials and methods in Text S1 for additional details.

### Biofilm disruption assay on plastic.

S. aureus biofilms were treated with antimicrobial agents, followed by enumeration of viable cell counts, as previously described (37). See the supplemental materials and methods in Text S1 for additional details.

### Membrane potential measurements.

S. aureus membrane potential was determined using the fluorescent dye DiOC_2_ as previously described (100, 101) with some modifications. See the supplemental materials and methods in Text S1 for additional details.

### Laurdan membrane fluidity analysis.

S. aureus membrane fluidity was determined by Laurdan generalized polarization (GP) as previously described (101, 102) with some modifications. See the supplemental materials and methods in Text S1 for additional details.

### Fatty acid methyl ester analysis.

Whole-cell direct fatty acid methyl ester (FAME) analysis of S. aureus pellets was performed by Microbial ID, Inc. (Newark, DE), as previously described (103). See the supplemental materials and methods in Text S1 for additional details.

## References

[1] Høiby N, Bjarnsholt T, Givskov M, Molin S, Ciofu O 2010 Antibiotic resistance of bacterial biofilms. Int J Antimicrob Agents 35:322–332. doi:10.1016/j.ijantimicag.2009.12.011. 20149602

[2] Bhattacharya M, Wozniak DJ, Stoodley P, Hall-Stoodley L 2015 Prevention and treatment of *Staphylococcus aureus* biofilms. Expert Rev Anti Infect Ther 13:1499–1516. doi:10.1586/14787210.2015.1100533. 26646248 PMC5142822

[3] Wu H, Moser C, Wang H-Z, Høiby N, Song Z-J 2015 Strategies for combating bacterial biofilm infections. Int J Oral Sci 7:1–7. doi:10.1038/ijos.2014.65. 25504208 PMC4817533

[4] Penesyan A, Gillings M, Paulsen IT 2015 Antibiotic discovery: combatting bacterial resistance in cells and in biofilm communities. Molecules 20:5286–5298. doi:10.3390/molecules20045286. 25812150 PMC6272253

[5] Campoccia D, Montanaro L, Arciola CR 2006 The significance of infection related to orthopedic devices and issues of antibiotic resistance. Biomaterials 27:2331–2339. doi:10.1016/j.biomaterials.2005.11.044. 16364434

[6] Harris JK, De Groote MA, Sagel SD, Zemanick ET, Kapsner R, Penvari C, Kaess H, Deterding RR, Accurso FJ, Pace NR 2007 Molecular identification of bacteria in bronchoalveolar lavage fluid from children with cystic fibrosis. Proc Natl Acad Sci U S A 104:20529–20533. doi:10.1073/pnas.0709804104. 18077362 PMC2154465

[7] Filkins LM, Hampton TH, Gifford AH, Gross MJ, Hogan DA, Sogin ML, Morrison HG, Paster BJ, O’Toole GA 2012 Prevalence of streptococci and increased polymicrobial diversity associated with cystic fibrosis patient stability. J Bacteriol 194:4709–4717. doi:10.1128/JB.00566-12. 22753064 PMC3415522

[8] Fodor AA, Klem ER, Gilpin DF, Elborn JS, Boucher RC, Tunney MM, Wolfgang MC 2012 The adult cystic fibrosis airway microbiota is stable over time and infection type, and highly resilient to antibiotic treatment of exacerbations. PLoS One 7:e45001. doi:10.1371/journal.pone.0045001. 23049765 PMC3458854

[9] Stressmann FA, Rogers GB, van der Gast CJ, Marsh P, Vermeer LS, Carroll MP, Hoffman L, Daniels TWV, Patel N, Forbes B, Bruce KD 2012 Long-term cultivation-independent microbial diversity analysis demonstrates that bacterial communities infecting the adult cystic fibrosis lung show stability and resilience. Thorax 67:867–873. doi:10.1136/thoraxjnl-2011-200932. 22707521

[10] Zhao J, Schloss PD, Kalikin LM, Carmody LA, Foster BK, Petrosino JF, Cavalcoli JD, VanDevanter DR, Murray S, Li JZ, Young VB, LiPuma JJ 2012 Decade-long bacterial community dynamics in cystic fibrosis airways. Proc Natl Acad Sci U S A 109:5809–5814. doi:10.1073/pnas.1120577109. 22451929 PMC3326496

[11] Lim YW, Schmieder R, Haynes M, Willner D, Furlan M, Youle M, Abbott K, Edwards R, Evangelista J, Conrad D, Rohwer F 2013 Metagenomics and metatranscriptomics: windows on CF-associated viral and microbial communities. J Cyst Fibros 12:154–164. doi:10.1016/j.jcf.2012.07.009. 22951208 PMC3534838

[12] Filkins LM, O’Toole GA 2015 Cystic fibrosis lung infections: polymicrobial, complex, and hard to treat. PLoS Pathog 11:e1005258. doi:10.1371/journal.ppat.1005258. 26719892 PMC4700991

[13] Giacometti A, Cirioni O, Schimizzi AM, Del Prete MS, Barchiesi F, D’Errico MM, Petrelli E, Scalise G 2000 Epidemiology and microbiology of surgical wound infections. J Clin Microbiol 38:918–922. 10655417 10.1128/jcm.38.2.918-922.2000 PMC86247

[14] Citron DM, Goldstein EJC, Merriam CV, Lipsky BA, Abramson MA 2007 Bacteriology of moderate-to-severe diabetic foot infections and in vitro activity of antimicrobial agents. J Clin Microbiol 45:2819–2828. doi:10.1128/JCM.00551-07. 17609322 PMC2045270

[15] Dowd SE, Sun Y, Secor PR, Rhoads DD, Wolcott BM, James GA, Wolcott RD 2008 Survey of bacterial diversity in chronic wounds using pyrosequencing, DGGE, and full ribosome shotgun sequencing. BMC Microbiol 8:43. doi:10.1186/1471-2180-8-43. 18325110 PMC2289825

[16] Post JC, Preston RA, Aul JJ, Larkins-Pettigrew M, Rydquist-White J, Anderson KW, Wadowsky RM, Reagan DR, Walker ES, Kingsley LA, Magit AE, Ehrlich GD 1995 Molecular analysis of bacterial pathogens in otitis media with effusion. JAMA 273:1598–1604. doi:10.1001/jama.1995.03520440052036. 7745773

[17] Hendolin PH, Markkanen A, Ylikoski J, Wahlfors JJ 1997 Use of multiplex PCR for simultaneous detection of four bacterial species in middle ear effusions. J Clin Microbiol 35:2854–2858. 9350746 10.1128/jcm.35.11.2854-2858.1997 PMC230074

[18] Ronald A 2003 The etiology of urinary tract infection: traditional and emerging pathogens. Dis Mon 49:71–82. doi:10.1067/mda.2003.8. 12601338

[19] Kline KA, Lewis AL 2016 Gram-positive uropathogens, polymicrobial urinary tract infection, and the emerging microbiota of the urinary tract. Microbiol Spectr 4 (2):UTI-0012-2012. doi:10.1128/microbiolspec.UTI-0012-2012. PMC4888879 27227294

[20] Lightbown JW 1954 An antagonist of streptomycin and dihydrostreptomycin produced by *Pseudomonas aeruginosa*. J Gen Microbiol 11:477–492. doi:10.1099/00221287-11-3-477. 13221769

[21] Shahidi A, Ellner PD 1969 Effect of mixed cultures on antibiotic susceptibility testing. Appl Microbiol 18:766–770. 4313167 10.1128/am.18.5.766-770.1969 PMC378086

[22] Barry AL, Joyce LJ, Adams AP, Benner EJ 1973 Rapid determination of antimicrobial susceptibility for urgent clinical situations. Am J Clin Pathol 59:693–699. doi:10.1093/ajcp/59.5.693. 4633996

[23] Ellner PD, Johnson E 1976 Unreliability of direct antibiotic susceptibility testing on wound exudates. Antimicrob Agents Chemother 9:355–356. doi:10.1128/aac.9.2.355. 1267433 PMC429528

[24] Hollick GE, Washington JA 1976 Comparison of direct and standardized disk diffusion susceptibility testing of urine cultures. Antimicrob Agents Chemother 9:804–809. doi:10.1128/aac.9.5.804. 949178 PMC429625

[25] Johnson JE, Washington JA 1976 Comparison of direct and standardized antimicrobial susceptibility testing of positive blood cultures. Antimicrob Agents Chemother 10:211–214. doi:10.1128/aac.10.2.211. 984763 PMC429722

[26] Linn BS, Szabo S 1975 The varying sensitivity to antibacterial agents of micro-organisms in pure vs. mixed cultures. Surgery 77:780–785. 806983

[27] Lebrun M, de Repentigny J, Mathieu LG 1978 [Diminution of the antibacterial activity of antibiotics in cultures and in experimental mixed infections.] Can J Microbiol 24:154–161. doi:10.1139/m78-028. 417782

[28] Mirrett S, Reller LB 1979 Comparison of direct and standard antimicrobial disk susceptibility testing for bacteria isolated from blood. J Clin Microbiol 10:482–487. 528681 10.1128/jcm.10.4.482-487.1979 PMC273200

[29] Hoffman LR, Deziel E, D’Argenio DA, Lepine F, Emerson J, McNamara S, Gibson RL, Ramsey BW, Miller SI 2006 Selection for *Staphylococcus aureus* small-colony variants due to growth in the presence of *Pseudomonas aeruginosa*. Proc Natl Acad Sci U S A 103:19890–19895. doi:10.1073/pnas.0606756104. 17172450 PMC1750898

[30] Ryan RP, Fouhy Y, Garcia BF, Watt SA, Niehaus K, Yang L, Tolker-Nielsen T, Dow JM 2008 Interspecies signalling via the *Stenotrophomonas maltophilia* diffusible signal factor influences biofilm formation and polymyxin tolerance in *Pseudomonas aeruginosa*. Mol Microbiol 68:75–86. doi:10.1111/j.1365-2958.2008.06132.x. 18312265

[31] Harriott MM, Noverr MC 2009 *Candida albicans* and *Staphylococcus aureus* form polymicrobial biofilms: effects on antimicrobial resistance. Antimicrob Agents Chemother 53:3914–3922. doi:10.1128/AAC.00657-09. 19564370 PMC2737866

[32] Armbruster CE, Hong W, Pang B, Weimer KED, Juneau RA, Turner J, Swords WE 2010 Indirect pathogenicity of *Haemophilus influenzae* and *Moraxella catarrhalis* in polymicrobial otitis media occurs via interspecies quorum signaling. mBio 1:e00102-10. doi:10.1128/mBio.00102-10. 20802829 PMC2925075

[33] Bernier SP, Létoffé S, Delepierre M, Ghigo J-M 2011 Biogenic ammonia modifies antibiotic resistance at a distance in physically separated bacteria. Mol Microbiol 81:705–716. doi:10.1111/j.1365-2958.2011.07724.x. 21651627

[34] Vega NM, Allison KR, Samuels AN, Klempner MS, Collins JJ 2013 *Salmonella typhimurium* intercepts *Escherichia coli* signaling to enhance antibiotic tolerance. Proc Natl Acad Sci U S A 110:14420–14425. doi:10.1073/pnas.1308085110. 23946425 PMC3761632

[35] DeLeon S, Clinton A, Fowler H, Everett J, Horswill AR, Rumbaugh KP 2014 Synergistic interactions of *Pseudomonas aeruginosa* and *Staphylococcus aureus* in an *in vitro* wound model. Infect Immun 82:4718–4728. doi:10.1128/IAI.02198-14. 25156721 PMC4249327

[36] Beaudoin T, Yau YCW, Stapleton PJ, Gong Y, Wang PW, Guttman DS, Waters V 2017 *Staphylococcus aureus* interaction with *Pseudomonas aeruginosa* biofilm enhances tobramycin resistance. NPJ Biofilms Microbiomes 3:25. doi:10.1038/s41522-017-0035-0. 29062489 PMC5648753

[37] Orazi G, O’Toole GA 2017 *Pseudomonas aeruginosa* alters *Staphylococcus aureus* sensitivity to vancomycin in a biofilm model of cystic fibrosis infection. mBio 8:e00873-17. doi:10.1128/mBio.00873-17. 28720732 PMC5516255

[38] Kean R, Rajendran R, Haggarty J, Townsend EM, Short B, Burgess KE, Lang S, Millington O, Mackay WG, Williams C, Ramage G 2017 *Candida albicans* mycofilms support *Staphylococcus aureus* colonization and enhances miconazole resistance in dual-species interactions. Front Microbiol 8:258. doi:10.3389/fmicb.2017.00258. 28280487 PMC5322193

[39] Radlinski L, Rowe SE, Kartchner LB, Maile R, Cairns BA, Vitko NP, Gode CJ, Lachiewicz AM, Wolfgang MC, Conlon BP 2017 *Pseudomonas aeruginosa* exoproducts determine antibiotic efficacy against *Staphylococcus aureus*. PLoS Biol 15:e2003981. doi:10.1371/journal.pbio.2003981. 29176757 PMC5720819

[40] Adamowicz EM, Flynn J, Hunter RC, Harcombe WR 2018 Cross-feeding modulates antibiotic tolerance in bacterial communities. ISME J 15:555. doi:10.1038/s41396-018-0212-z. PMC6194032 29991761

[41] Cystic Fibrosis Foundation. 2015 Cystic Fibrosis Foundation patient registry 2015 annual data report. Cystic Fibrosis Foundation, Bethesda, MD.

[42] Cox MJ, Allgaier M, Taylor B, Baek MS, Huang YJ, Daly RA, Karaoz U, Andersen GL, Brown R, Fujimura KE, Wu B, Tran D, Koff J, Kleinhenz ME, Nielson D, Brodie EL, Lynch SV 2010 Airway microbiota and pathogen abundance in age-stratified cystic fibrosis patients. PLoS One 5:e11044. doi:10.1371/journal.pone.0011044. 20585638 PMC2890402

[43] Wolter DJ, Emerson JC, McNamara S, Buccat AM, Qin X, Cochrane E, Houston LS, Rogers GB, Marsh P, Prehar K, Pope CE, Blackledge M, Deziel E, Bruce KD, Ramsey BW, Gibson RL, Burns JL, Hoffman LR 2013 *Staphylococcus aureus* small-colony variants are independently associated with worse lung disease in children with cystic fibrosis. Clin Infect Dis 57:384–391. doi:10.1093/cid/cit270. 23625938 PMC3888146

[44] Emerson J, Rosenfeld M, McNamara S, Ramsey B, Gibson RL 2002 *Pseudomonas aeruginosa* and other predictors of mortality and morbidity in young children with cystic fibrosis. Pediatr Pulmonol 34:91–100. doi:10.1002/ppul.10127. 12112774

[45] Com G, Carroll JL, Castro MM, Tang X, Jambhekar S, Berlinski A 2014 Predictors and outcome of low initial forced expiratory volume in 1 second measurement in children with cystic fibrosis. J Pediatr 164:832–838. doi:10.1016/j.jpeds.2013.11.064. 24418473

[46] Hudson VL, Wielinski CL, Regelmann WE 1993 Prognostic implications of initial oropharyngeal bacterial flora in patients with cystic fibrosis diagnosed before the age of two years. J Pediatr 122:854–860. doi:10.1016/s0022-3476(09)90007-5. 8501559

[47] Rosenbluth DB, Wilson K, Ferkol T, Schuster DP 2004 Lung function decline in cystic fibrosis patients and timing for lung transplantation referral. Chest 126:412–419. doi:10.1378/chest.126.2.412. 15302726

[48] Limoli DH, Yang J, Khansaheb MK, Helfman B, Peng L, Stecenko AA, Goldberg JB 2016 *Staphylococcus aureus* and *Pseudomonas aeruginosa* co-infection is associated with cystic fibrosis-related diabetes and poor clinical outcomes. Eur J Clin Microbiol Infect Dis 35:947–953. doi:10.1007/s10096-016-2621-0. 26993289

[49] Maliniak ML, Stecenko AA, McCarty NA 2016 A longitudinal analysis of chronic MRSA and *Pseudomonas aeruginosa* co-infection in cystic fibrosis: a single-center study. J Cyst Fibros 15:350–356. doi:10.1016/j.jcf.2015.10.014. 26610860

[50] Limoli DH, Hoffman LR 2019 Help, hinder, hide and harm: what can we learn from the interactions between *Pseudomonas aeruginosa* and *Staphylococcus aureus* during respiratory infections? Thorax 74:684–692. doi:10.1136/thoraxjnl-2018-212616. 30777898 PMC6585302

[51] Gjødsbøl K, Christensen JJ, Karlsmark T, Jørgensen B, Klein BM, Krogfelt KA 2006 Multiple bacterial species reside in chronic wounds: a longitudinal study. Int Wound J 3:225–231. doi:10.1111/j.1742-481X.2006.00159.x. 16984578 PMC7951738

[52] Körber A, Schmid EN, Buer J, Klode J, Schadendorf D, Dissemond J 2010 Bacterial colonization of chronic leg ulcers: current results compared with data 5 years ago in a specialized dermatology department. J Eur Acad Dermatol Venereol 24:1017–1025. doi:10.1111/j.1468-3083.2010.03570.x. 20236200

[53] Pastar I, Nusbaum AG, Gil J, Patel SB, Chen J, Valdes J, Stojadinovic O, Plano LR, Tomic-Canic M, Davis SC 2013 Interactions of methicillin resistant *Staphylococcus aureus* USA300 and *Pseudomonas aeruginosa* in polymicrobial wound infection. PLoS One 8:e56846. doi:10.1371/journal.pone.0056846. 23451098 PMC3579943

[54] Silva MT, Sousa JC, Macedo MA, Polónia J, Parente AM 1976 Effects of phenethyl alcohol on *Bacillus* and *Streptococcus*. J Bacteriol 127:1359–1369. 60333 10.1128/jb.127.3.1359-1369.1976 PMC232931

[55] Heipieper HJ, Keweloh H, Rehm HJ 1991 Influence of phenols on growth and membrane permeability of free and immobilized *Escherichia coli*. Appl Environ Microbiol 57:1213–1217. 2059043 10.1128/aem.57.4.1213-1217.1991 PMC182870

[56] McDonnell G, Russell AD 1999 Antiseptics and disinfectants: activity, action, and resistance. Clin Microbiol Rev 12:147–179. doi:10.1128/CMR.12.1.147. 9880479 PMC88911

[57] Miller MH, Edberg SC, Mandel LJ, Behar CF, Steigbigel NH 1980 Gentamicin uptake in wild-type and aminoglycoside-resistant small-colony mutants of *Staphylococcus aureus*. Antimicrob Agents Chemother 18:722–729. doi:10.1128/aac.18.5.722. 7447428 PMC284082

[58] Tsuji BT, Eiff von C, Kelchlin PA, Forrest A, Smith PF 2008 Attenuated vancomycin bactericidal activity against *Staphylococcus aureus hemB* mutants expressing the small-colony-variant phenotype. Antimicrob Agents Chemother 52:1533–1537. doi:10.1128/AAC.01254-07. 18285476 PMC2292514

[59] Hess DJ, Henry-Stanley MJ, Lusczek ER, Beilman GJ, Wells CL 2013 Anoxia inhibits biofilm development and modulates antibiotic activity. J Surg Res 184:488–494. doi:10.1016/j.jss.2013.04.049. 23746961

[60] Proctor RA, von Humboldt A 1998 Bacterial energetics and antimicrobial resistance. Drug Resist Updat 1:227–235. doi:10.1016/S1368-7646(98)80003-4. 16904405

[61] Hurdle JG, O’Neill AJ, Chopra I, Lee RE 2011 Targeting bacterial membrane function: an underexploited mechanism for treating persistent infections. Nat Rev Microbiol 9:62–75. doi:10.1038/nrmicro2474. 21164535 PMC3496266

[62] von Eiff C, Heilmann C, Proctor RA, Woltz C, Peters G, Götz F 1997 A site-directed *Staphylococcus aureus hemB* mutant is a small-colony variant which persists intracellularly. J Bacteriol 179:4706–4712. doi:10.1128/jb.179.15.4706-4712.1997. 9244256 PMC179315

[63] Lepine F, Deziel E, Milot S, Rahme LG 2003 A stable isotope dilution assay for the quantification of the *Pseudomonas* quinolone signal in *Pseudomonas aeruginosa* cultures. Biochim Biophys Acta 1622:36–41. doi:10.1016/S0304-4165(03)00103-X. 12829259

[64] Deziel E, Lépine F, Milot S, He J, Mindrinos MN, Tompkins RG, Rahme LG 2004 Analysis of *Pseudomonas aeruginosa* 4-hydroxy-2-alkylquinolines (HAQs) reveals a role for 4-hydroxy-2-heptylquinoline in cell-to-cell communication. Proc Natl Acad Sci U S A 101:1339–1344. doi:10.1073/pnas.0307694100. 14739337 PMC337054

[65] Lightbown JW, Jackson FL 1956 Inhibition of cytochrome systems of heart muscle and certain bacteria by the antagonists of dihydrostreptomycin: 2-alkyl-4-hydroxyquinoline N-oxides. Biochem J 63:130–137. doi:10.1042/bj0630130. 13315258 PMC1216010

[66] Van Ark G, Berden JA 1977 Binding of HQNO to beef-heart sub-mitochondrial particles. Biochim Biophys Acta 459:119–137. doi:10.1016/0005-2728(77)90014-7. 831781

[67] Esposti MD 1989 Prediction and comparison of the haem-binding sites in membrane haemoproteins. Biochim Biophys Acta 977:249–265. doi:10.1016/S0005-2728(89)80079-9. 2686753

[68] Miyadera H, Shiomi K, Ui H, Yamaguchi Y, Masuma R, Tomoda H, Miyoshi H, Osanai A, Kita K, Omura S 2003 Atpenins, potent and specific inhibitors of mitochondrial complex II (succinate-ubiquinone oxidoreductase). Proc Natl Acad Sci U S A 100:473–477. doi:10.1073/pnas.0237315100. 12515859 PMC141019

[69] Keweloh H, Diefenbach R, Rehm H-J 1991 Increase of phenol tolerance of *Escherichia coli* by alterations of the fatty acid composition of the membrane lipids. Arch Microbiol 157:49–53. 1814276 10.1007/BF00245334

[70] Domenech O, Dufrêne YF, Van Bambeke F, Tukens PM, Mingeot-Leclercq M-P 2010 Interactions of oritavancin, a new semi-synthetic lipoglycopeptide, with lipids extracted from *Staphylococcus aureus*. Biochim Biophys Acta 1798:1876–1885. doi:10.1016/j.bbamem.2010.06.011. 20599683

[71] Bessa LJ, Ferreira M, Gameiro P 2018 Evaluation of membrane fluidity of multidrug-resistant isolates of *Escherichia coli* and *Staphylococcus aureus* in presence and absence of antibiotics. J Photochem Photobiol B 181:150–156. doi:10.1016/j.jphotobiol.2018.03.002. 29567316

[72] Perez-Lopez MI, Mendez-Reina R, Trier S, Herrfurth C, Feussner I, Bernal A, Forero-Shelton M, Leidy C 2019 Variations in carotenoid content and acyl chain composition in exponential, stationary and biofilm states of Staphylococcus *aureus*, and their influence on membrane biophysical properties. Biochim Biophys Acta Biomembr 1861:978–987. doi:10.1016/j.bbamem.2019.02.001. 30771288

[73] Friedlander G, Le Grimellec C, Giocondi M-C, Amiel C 1987 Benzyl alcohol increases membrane fluidity and modulates cyclic AMP synthesis in intact renal epithelial cells. Biochim Biophys Acta Biomembr 903:341–348. doi:10.1016/0005-2736(87)90224-0. 2820491

[74] Shigapova N, Török Z, Balogh G, Goloubinoff P, Vígh L, Horváth I 2005 Membrane fluidization triggers membrane remodeling which affects the thermotolerance in *Escherichia coli*. Biochem Biophys Res Commun 328:1216–1223. doi:10.1016/j.bbrc.2005.01.081. 15708006

[75] Cebrián G, Condón S, Mañas P 2016 Influence of growth and treatment temperature on *Staphylococcus aureus* resistance to pulsed electric fields: relationship with membrane fluidity. Innov Food Sci Emerg Technol 37:161–169. doi:10.1016/j.ifset.2016.08.011.

[76] Balogh G, Horváth I, Nagy E, Hoyk Z, Benkõ S, Bensaude O, Vígh L 2005 The hyperfluidization of mammalian cell membranes acts as a signal to initiate the heat shock protein response. FEBS J 272:6077–6086. doi:10.1111/j.1742-4658.2005.04999.x. 16302971

[77] Veerman ECI, Valentijn-Benz M, Nazmi K, Ruissen ALA, Walgreen-Weterings E, van Marle J, Doust AB, van’t Hof W, Bolscher JGM, Amerongen A 2007 Energy depletion protects *Candida albicans* against antimicrobial peptides by rigidifying its cell membrane. J Biol Chem 282:18831–18841. doi:10.1074/jbc.M610555200. 17485465

[78] Daeschlein G 2013 Antimicrobial and antiseptic strategies in wound management. Int Wound J 10(Suppl 1):9–14. doi:10.1111/iwj.12175. 24251838 PMC7950476

[79] Assadian O 2016 Octenidine dihydrochloride: chemical characteristics and antimicrobial properties. J Wound Care 25:S3–S6. doi:10.12968/jowc.2016.25.Sup3.S3. 26949863

[80] Barr HL, Halliday N, Cámara M, Barrett DA, Williams P, Forrester DL, Simms R, Smyth AR, Honeybourne D, Whitehouse JL, Nash EF, Dewar J, Clayton A, Knox AJ, Fogarty AW 2015 *Pseudomonas aeruginosa* quorum sensing molecules correlate with clinical status in cystic fibrosis. Eur Respir J 46:1046–1054. doi:10.1183/09031936.00225214. 26022946 PMC4589431

[81] Smith EE, Buckley DG, Wu Z, Saenphimmachak C, Hoffman LR, D’Argenio DA, Miller SI, Ramsey BW, Speert DP, Moskowitz SM, Burns JL, Kaul R, Olson MV 2006 Genetic adaptation by *Pseudomonas aeruginosa* to the airways of cystic fibrosis patients. Proc Natl Acad Sci U S A 103:8487–8492. doi:10.1073/pnas.0602138103. 16687478 PMC1482519

[82] Hoffman LR, Kulasekara HD, Emerson J, Houston LS, Burns JL, Ramsey BW, Miller SI 2009 *Pseudomonas aeruginosa lasR* mutants are associated with cystic fibrosis lung disease progression. J Cyst Fibros 8:66–70. doi:10.1016/j.jcf.2008.09.006. 18974024 PMC2631641

[83] Limoli DH, Whitfield GB, Kitao T, Ivey ML, Davis MR, Grahl N, Hogan DA, Rahme LG, Howell PL, O’Toole GA, Goldberg JB 2017 *Pseudomonas aeruginosa* alginate overproduction promotes coexistence with *Staphylococcus aureus* in a model of cystic fibrosis respiratory infection. mBio 8:e00186-17. doi:10.1128/mBio.00186-17. 28325763 PMC5362032

[84] Filkins LM, Graber JA, Olson DG, Dolben EL, Lynd LR, Bhuju S, O’Toole GA 2015 Coculture of *Staphylococcus aureus* with *Pseudomonas aeruginosa* drives *S. aureus* towards fermentative metabolism and reduced viability in a cystic fibrosis model. J Bacteriol 197:2252–2264. doi:10.1128/JB.00059-15. 25917910 PMC4524177

[85] Fuchs S, Pané-Farré J, Kohler C, Hecker M, Engelmann S 2007 Anaerobic gene expression in *Staphylococcus aureus*. J Bacteriol 189:4275–4289. doi:10.1128/JB.00081-07. 17384184 PMC1913399

[86] Burkhart BM, Li N, Langs DA, Pangborn WA, Duax WL 1998 The conducting form of gramicidin A is a right-handed double-stranded double helix. Proc Natl Acad Sci U S A 95:12950–12955. doi:10.1073/pnas.95.22.12950. 9789021 PMC23667

[87] Koo SP, Bayer AS, Yeaman MR 2001 Diversity in antistaphylococcal mechanisms among membrane-targeting antimicrobial peptides. Infect Immun 69:4916–4922. doi:10.1128/IAI.69.8.4916-4922.2001. 11447168 PMC98582

[88] Xiong YQ, Mukhopadhyay K, Yeaman MR, Adler-Moore J, Bayer AS 2005 Functional interrelationships between cell membrane and cell wall in antimicrobial peptide-mediated killing of *Staphylococcus aureus*. Antimicrob Agents Chemother 49:3114–3121. doi:10.1128/AAC.49.8.3114-3121.2005. 16048912 PMC1196293

[89] Kristiansen JE, Amaral L 1997 The potential management of resistant infections with non-antibiotics. J Antimicrob Chemother 40:319–327. doi:10.1093/jac/40.3.319. 9338482

[90] Mazumder R, Ganguly K, Dastidar SG, Chakrabarty AN 2001 Trifluoperazine: a broad spectrum bactericide especially active on staphylococci and vibrios. Int J Antimicrob Agents 18:403–406. doi:10.1016/S0924-8579(01)00324-7. 11691578

[91] Kristiansen JE, Hendricks O, Delvin T, Butterworth TS, Aagaard L, Christensen JB, Flores VC, Keyzer H 2007 Reversal of resistance in microorganisms by help of non-antibiotics. J Antimicrob Chemother 59:1271–1279. doi:10.1093/jac/dkm071. 17403708

[92] Kristiansen JE, Thomsen VF, Martins A, Viveiros M, Amaral L 2010 Non-antibiotics reverse resistance of bacteria to antibiotics. In Vivo 24:751–754. 20952744

[93] Mandal A, Sinha C, Kumar Jena A, Ghosh S, Samanta A 2010 An investigation on *in vitro* and *in vivo* antimicrobial properties of the antidepressant: amitriptyline hydrochloride. Braz J Microbiol 41:635–645. doi:10.1590/S1517-83822010000300014. 24031539 PMC3768647

[94] Spitzer M, Griffiths E, Blakely KM, Wildenhain J, Ejim L, Rossi L, De Pascale G, Curak J, Brown E, Tyers M, Wright GD 2011 Cross-species discovery of syncretic drug combinations that potentiate the antifungal fluconazole. Mol Syst Biol 7:499–499. doi:10.1038/msb.2011.31. 21694716 PMC3159983

[95] Caetano W, Tabak M 1999 Interaction of chlorpromazine and trifluoperazine with ionic micelles: electronic absorption spectroscopy studies. Spectrochim Acta A Mol Biomol Spectrosc 55:2513–2528. doi:10.1016/S1386-1425(99)00043-8.

[96] Hendrich AB, Wesołowska O, Michalak K 2001 Trifluoperazine induces domain formation in zwitterionic phosphatidylcholine but not in charged phosphatidylglycerol bilayers. Biochim Biophys Acta 1510:414–425. doi:10.1016/S0005-2736(00)00373-4. 11342176

[97] Kalani M, Brismar K, Fagrell B, Ostergren J, Jorneskog G 1999 Transcutaneous oxygen tension and toe blood pressure as predictors for outcome of diabetic foot ulcers. Diab Care 22:147–151. doi:10.2337/diacare.22.1.147. 10333917

[98] Wattel F, Mathieu D, Coget JM, Billard V 1990 Hyperbaric oxygen therapy in chronic vascular wound management. Angiology 41:59–65. doi:10.1177/000331979004100109. 2306000

[99] Kopf SH, Sessions AL, Cowley ES, Reyes C, Van Sambeek L, Hu Y, Orphan VJ, Kato R, Newman DK 2016 Trace incorporation of heavy water reveals slow and heterogeneous pathogen growth rates in cystic fibrosis sputum. Proc Natl Acad Sci U S A 113:E110–E116. doi:10.1073/pnas.1512057112. 26715741 PMC4720290

[100] Nair DR, Monteiro JM, Memmi G, Thanassi J, Pucci M, Schwartzman J, Pinho MG, Cheung AL 2015 Characterization of a novel small molecule that potentiates β-lactam activity against gram-positive and gram-negative pathogens. Antimicrob Agents Chemother 59:1876–1885. doi:10.1128/AAC.04164-14. 25583731 PMC4356822

[101] Müller A, Wenzel M, Strahl H, Grein F, Saaki TNV, Kohl B, Siersma T, Bandow JE, Sahl H-G, Schneider T, Hamoen LW 2016 Daptomycin inhibits cell envelope synthesis by interfering with fluid membrane microdomains. Proc Natl Acad Sci U S A 113:E7077–E7086. doi:10.1073/pnas.1611173113. 27791134 PMC5111643

[102] Strahl H, Bürmann F, Hamoen LW 2014 The actin homologue MreB organizes the bacterial cell membrane. Nat Commun 5:3442. doi:10.1038/ncomms4442. 24603761 PMC3955808

[103] Zhu K, Bayles DO, Xiong A, Jayaswal RK, Wilkinson BJ 2005 Precursor and temperature modulation of fatty acid composition and growth of *Listeria monocytogenes* cold-sensitive mutants with transposon-interrupted branched-chain-keto acid dehydrogenase. Microbiology 151:615–623. doi:10.1099/mic.0.27634-0. 15699210

