# Supplementary material for: Pseudomonas aeruginosa Increases the Sensitivity of Biofilm-Grown Staphylococcus aureus to Membrane-Targeting Antiseptics and Antibiotics
Source: mBio. 2019 Jul 30;10(4):e01501-19. doi: 10.1128/mBio.01501-19 (PMC6667622; doi:10.1128/mBio.01501-19)
Supplement: TABLE S1 [file mBio.01501-19-st001.pdf]

Table S1. ***P. aeruginosa*** **supernatant increases** ***S. aureus*** **sensitivity to antiseptics and antibiotics.**

Mechanism of action	Class	Compound name
Membrane-active compounds	Antiseptic / disinfectant; phenol	**Chloroxylenol *** **Triclosan *** **2-Phenylphenol *** Pentachlorophenol †
	Antiseptic / disinfectant; biguanide	Alexidine # Chlorhexidine #
	Polymyxin	Polymyxin B # Colistin #
Nucleic acid synthesis inhibitors	Sulfonamide	Sulfadiazine # Sulfamethazine † Sulfamethoxazole # Sulfathiazole † Sulfisoxazole #
	Fluoroquinolone	Ciprofloxacin # Ofloxacin # **Norfloxacin ***
	First generation quinolone	Pipemidic acid # Enoxacin #
	Anaerobic DNA synthesis inhibitor	Ornidazole # Nitrofurantoin # Tinidazole †
	Fungicide; RNA synthesis inhibitor	Oxycarboxin †
	Antiseptic	Proflavine †
	Antiviral; DNA synthesis inhibitor	Trifluorothymidine †
Protein synthesis inhibitors	Aminoglycoside	Apramycin # Dihydrostreptomycin † Spectinomycin †

	Tetracycline	Demeclocycline #<br>Oxytetracycline †
	Amphenicol	Thiamphenicol #
	Macrolide	Troleandomycin #<br>Blasticidin S †
Cell wall synthesis inhibitors	Beta-lactam	Aztreonam #<br>Cefoperazone †<br>Cefotaxime †<br>Piperacillin †
Metal chelators	Bipyridine	2,2'-Dipyridyl #
	Semicarbazide	Nitrofurazone †<br>Semicarbazide †<br>Semicarbazone †
	Antiseptic; phenol	8-Hydroxyquinoline #
Proton ionophores	Antiseptic	2,4-Dinitrophenol †<br>FCCP †
Non-traditional antibiotics	Anesthetic; Na+ channel blocker	Lidocaine #<br>Procaine #<br>Promethazine #
	Parkinson's treatment; Anticholinergic	**Amitriptyline***<br>Atropine †<br>Orphenadrine #<br>Pridinol #
	Parkinson's treatment; Aromatic L-amino acid decarboxylase	Benserazide #
	Alcoholism treatment; acetaldehyde dehydrogenase inhibitor	Disulfiram #
	Nonsteroidal anti-inflammatory drug	Ketoprofen #

Phenylpropanoid	Cinnamic acid #<br>Coumarin #<br>4-Hydroxycoumarin #<br>Umbelliferone †
Triazole	Guanazole #
Antipsychotic; phenothiazine	Chlorpromazine #<br>**Trifluoperazine ***
β -adrenergic receptor blocker	D,L-Propanolol #
Nitrofuran	Furaltadone #
Naphthoquinone derivative	Plumbagin #
Other	Aluminum sulfate †<br>9-Aminoacridine †<br>4-Aminopyridine †<br>5-Azacytidine †<br>Azathioprine †<br>Captan †<br>Cefamandole naftate †<br>Cesium chloride †<br>Chlorambucil †<br>Cobalt chloride †<br>Cupric chloride †<br>Dichlofluanid †<br>3,5-Dinitrobenzene †<br>Dodine †<br>EGTA †<br>Ferric chloride †<br>Fusaric acid †<br>Gallic acid †<br>L-Glutamic-g-hydroxamate †<br>Harmane †<br>Hexammine cobalt (III) chloride †<br>Hydroxylamine †<br>Hygromycin B †<br>Iodoacetate †<br>Lauryl sulfobetaine †<br>Methyltrioctyl-ammonium chloride †<br>Nickel chloride †

PMSF †
Potassium tellurite †
D,L-Serine hydroxamate †
Sodium azide †
Sodium bromate †
Sodium cyanate †
Sodium metasilicate †
Sodium m-periodate †
Sodium nitrite †
Sodium pyrophosphate decahydrate †
Sodium salicylate †
Sodium selenite †
Sorbic acid †
Thallium (I) acetate †
Thioglycerol †
Tolylfluanid †
Zinc chloride †

Reported are compounds that became more effective at killing *S. aureus* biofilms when in the presence of *P. aeruginosa* exoproducts following a screen of Biolog Phenotype MicroArray Bacterial Chemical Sensitivity Panels. Increased efficacy of a drug was defined as at least a 10-fold decrease in CFU between *S. aureus* exposed to the antibiotic alone and *S. aureus* exposed to *P. aeruginosa* supernatant plus the antibiotic.

* Compounds in bold and marked with an asterisk were shown to have greater efficacy in the presence of *P. aeruginosa* supernatant against pre-formed (6 h) *S. aureus* biofilms.

# Compounds that did not have increased efficacy in the presence of *P. aeruginosa* supernatant against pre-formed (6 h) *S. aureus* biofilms.

† Compounds that have not been validated against pre-formed (6 h) biofilms, either due to lack of commercial availability, high toxicity, or because other representatives from the same class were tested.
