# Supplementary material for: Pseudomonas aeruginosa Increases the Sensitivity of Biofilm-Grown Staphylococcus aureus to Membrane-Targeting Antiseptics and Antibiotics
Source: mBio. 2019 Jul 30;10(4):e01501-19. doi: 10.1128/mBio.01501-19 (PMC6667622; doi:10.1128/mBio.01501-19)
Supplement: TEXT S1 [file mBio.01501-19-s0001.pdf]

Supplemental Text

Supplemental Results

HQNO likely does not increase *S. aureus* sensitivity to chloroxylenol via inhibition of the electron transport chain. Among the chemical inhibitors of electron transport tested, the only ones that altered *S. aureus* susceptibility to chloroxylenol are Antimycin A, and to a lesser extent, oligomycin (Fig. S3B, D). Only exposure to Antimycin A (Fig. S3B) fully recapitulated the effect of HQNO (Fig. 3C). However, it is still controversial as to whether Antimycin A inhibits Complex III of the *S. aureus* ETC (1, 2); the concentrations of Antimycin A tested in previous studies (0.7 and 1.4 μg/ml) were much lower than those used in our experiments (ranging from 3.7 to 100 μg/ml). Thus, it is currently unknown whether the concentrations of Antimycin A we used inhibit *S. aureus* ETC transport.

Dissipation of the proton motive force does not alter *S. aureus* susceptibility to chloroxylenol. Flow through the ETC generates proton motive force (PMF). Therefore, a consequence of ETC inhibition is the dissipation of the PMF. Here, we tested whether HQNO-mediated ETC inhibition increases *S. aureus* antibiotic sensitivity via depletion of the PMF. First, we determined whether HQNO and Antimycin A cause a reduction in the transmembrane electric potential, one of the components of the PMF. Subsequently, we asked whether manipulating the PMF alters *S. aureus* sensitivity to chloroxylenol.

Transmembrane electric potential was measured using the fluorescent dye $DiOC_2$ (3, 4) following exposure of *S. aureus* Newman to either HQNO or Antimycin A. In addition, the proton ionophore carbonyl cyanide 3-chlorophenylhydrazone (CCCP), an inhibitor of PMF, was

used as a positive control, and demonstrated a reduction in membrane potential in our assay system. Following exposure to the above conditions for 24 h, *S. aureus* Newman cells grown in either medium alone or exposed to supernatant from the *P. aeruginosa* PA14 Δ*pqsL* Δ*pvdA* Δ*pchE* (the ΔΔΔ mutant) maintained high membrane potential (Fig. S3F). In contrast, membrane potential was depleted in cells exposed to either *P. aeruginosa* PA14 wild-type supernatant, HQNO, or Antimycin A (Fig. S3F). These results demonstrate that HQNO causes a substantial decrease in the *S. aureus* membrane potential. Interestingly, Antimycin A at a concentration of 100 μg/ml caused a reduction in the membrane potential, which is consistent with its potential ability to inhibit the *S. aureus* ETC.

Next, we investigated whether inhibiting the PMF enhances the ability of chloroxylenol to kill *S. aureus* Newman biofilms. Addition of CCCP at the same concentration used in the experiment above (5 μM) or a higher concentration (25 μM) did not increase the sensitivity of *S. aureus* Newman biofilms to chloroxylenol (Fig. S3G), suggesting that dissipation of the PMF does not enhance the anti-staphylococcal activity of this drug.

Reactive oxygen species do not increase *S. aureus* biofilm sensitivity to chloroxylenol. Given our data above, it is possible that HQNO causes an increase in *S. aureus* sensitivity to chloroxylenol by inhibiting the electron transport chain (ETC). It has been reported that HQNO-mediated inhibition of the *P. aeruginosa* ETC results in the generation of reactive oxygen species (ROS) (5). It is possible that HQNO similarly induces ROS formation in *S. aureus*, which in combination with the antibacterial action of chloroxylenol may lead to decreased viability. Thus, we tested whether production of ROS would hypersensitize *S. aureus* to

chloroxylenol. Exposure to menadione or hydrogen peroxide did not increase *S. aureus* Newman biofilm sensitivity to chloroxylenol (Fig. S4A, B). Furthermore, treatment with the ROS scavenger N-acetyl cysteine (NAC) did not protect *S. aureus* Newman biofilms from *P. aeruginosa* supernatant-mediated increase in sensitivity to chloroxylenol (Fig. S4C). We verified that the concentration of NAC used in our experiments (1 mg/ml) protects *S. aureus* Newman biofilms from ROS generated by menadione and hydrogen peroxide (Fig. S4D, E). Additionally as described above, *P. aeruginosa* supernatant increases *S. aureus* susceptibility to chloroxylenol under anoxic conditions (Fig. 2A). Together, these results suggest that ROS generation is not playing a role in modulating sensitivity to this antibiotic.

Evaluating the role of antibiotic transporters in *S. aureus* sensitivity to chloroxylenol. In a previous study from our group, we examined changes in *S. aureus* gene expression induced by co-culture with *P. aeruginosa* by RNA-seq (6). We observed that *S. aureus* downregulates multiple known and putative antibiotic transporters when in the presence of *P. aeruginosa* (6). Thus, we hypothesized that a *P. aeruginosa*-dependent downregulation of drug efflux could lead to enhanced susceptibility of *S. aureus* to chloroxylenol we observe. To examine this possibility, we tested whether *S. aureus* JE2 strains with transposon insertions in genes encoding these antibiotic transporters exhibited increased sensitivity to chloroxylenol (Table S3; mutants denoted by *). None of the mutants tested were hypersensitive to chloroxylenol compared to the wild-type JE2 strain, suggesting that none of the transporters examined above is alone mediating *S. aureus* resistance to chloroxylenol. As in the case of other antibiotics and biocides, it is possible that several different transporters contribute to the efflux of chloroxylenol; thus, the

absence of a single transporter may not have an appreciable effect on sensitivity of *S. aureus* to the antibiotic.

Manipulating *S. aureus* fatty acid composition impacts sensitivity to chloroxylenol. We showed that increased membrane fluidity leads to increased *S. aureus* sensitivity to chloroxylenol. Thus, we hypothesized that manipulating the lipid profile of the *S. aureus* cell membrane to increase membrane fluidity – either via providing exogenous unsaturated fatty acids or using mutants that have higher levels of branched-chain fatty acids (BCFAs) relative to short chain fatty acids (SCFAs) – would increase the susceptibility of *S. aureus* biofilms to this antimicrobial compound.

First, we examined *S. aureus* biofilm sensitivity to chloroxylenol upon exposure to various fatty acids. *S. aureus* can incorporate exogenous unsaturated fatty acids into the cell membrane (7, 8), and it has been shown that the incorporation of unsaturated fatty acids into bacterial membranes increases membrane fluidity (9). We observed that the addition of unsaturated fatty acids increased *S. aureus* Newman biofilm sensitivity to chloroxylenol, and furthermore, a higher level of unsaturation (greater number of double bonds, and thus increased membrane fluidity) corresponded to a greater enhancement of antibiotic sensitivity (Fig. S5A). In contrast, exposure to stearic acid, a saturated fatty acid with the same number of carbons, did not enhance chloroxylenol's activity against *S. aureus* Newman (Fig. S5A). These findings support our model that an increase in membrane fluidity enhances the efficacy of the antimicrobial agent chloroxylenol against *S. aureus* biofilms.

High levels of BCFAs in bacterial cell membranes, especially the anteiso configuration, cause an increase in membrane fluidity (10-13). Thus, we tested whether shifting the proportion of *S. aureus* branched-chain fatty acids relative to SCFAs alters *S. aureus* biofilm sensitivity to chloroxylenol. We tested *S. aureus* USA300 strains with mutations in genes encoding branched-chain amino acid (BCAA) transporters, as leucine, isoleucine, and valine are used for the production of anteiso- and iso-BCFAs (14). When grown in a chemically-defined medium, the *S. aureus* USA300 Δ*brnQ1* mutant was shown to have a high proportion of BCFAs relative to SCFAs, that were entirely anteiso configuration (14); this fatty acid profile is associated with high membrane fluidity. For each BCAA transporter mutant tested, there was a trend towards higher sensitivity to chloroxylenol compared to the parental strain, although these changes were not statistically significant (Fig. S5B).

Here, we examined the impact of decreasing the proportion of *S. aureus* BCFAs on sensitivity to chloroxylenol. We tested a *S. aureus* SH1000 strain with a mutation in the *lpd* gene, which encodes the enzyme dihydrolipoamide dehydrogenase, a component of the branched-chain-keto acid dehydrogenase complex. The SH1000 *lpd* mutant is deficient in the early steps of BCFA synthesis, and consequently has reduced levels of BCFAs in the cell membrane, and lower membrane fluidity relative to the parental strain (13). This mutant is not hypersensitive to chloroxylenol compared to the parental strain (Fig. S5C).

Finally, we examined whether changes in *S. aureus* phospholipid headgroup profiles could explain the observed changes in antibiotic sensitivity. Exposure to hypoxia leads to increased amounts of cardiolipin (CL) in *B. subtilis* (15). Furthermore, CL has been shown to decrease the

packing of lipid bilayers; high CL levels corresponded to an increase in fluidity (16). Therefore, one potential explanation for the HQNO-mediated shift in fluidity is an increase in the level of CL in the membrane. To test this hypothesis, we assessed whether *P. aeruginosa* supernatant could still increase the antibiotic sensitivity of a *S. aureus* JE2 strain lacking the ability to synthesize CL. We found that strains with mutations in the *cls* gene, which encodes cardiolipin synthase, exhibited a *P. aeruginosa* supernatant-mediated increase in antibiotic sensitivity to levels similar to the parental strain (Fig. S5D). This result suggests that the presence of CL is not required for the observed HQNO-mediated increase in *S. aureus* sensitivity to chloroxylenol.

HQNO alters *S. aureus* membrane fatty acid profiles. Over the course of 10 h, BCFA levels increased steadily over time relative to the proportion of SCFAs in *S. aureus* Newman exposed to medium alone, reaching 91.3% of the fatty acid pool by 10 h, whereas the proportion of BCFAs in the membrane of *P. aeruginosa* supernatant-treated cells remained fairly constant (78.5% at 10 h, Fig. S6A, Table S2). Furthermore, cells exposed to *P. aeruginosa* supernatant maintained fairly constant levels of anteiso- and iso-BCFAs (Fig. S6B), which is reflected by the stable anteiso/iso ratio (Fig. S6C). In contrast, the proportion of anteiso-BCFAs relative to iso-BCFAs increased steadily over time in the MEM-exposed control cells (Fig. S6B, C). Together, we observed that while *S. aureus* Newman growing in MEM shifted its fatty acid profile over time, *S. aureus* exposed to *P. aeruginosa* supernatant maintained fairly constant fatty acid levels.

Additionally, we performed an experiment similar to the one above in which we grew *S. aureus* in shaking flasks for 24 h under different conditions: *S. aureus* Newman was exposed to either medium (MEM) alone, *P. aeruginosa* PA14 supernatants (from wild-type or the Δ*pqsL* Δ*pvdA*

Δ*pchE* mutant), HQNO, or Antimycin A, and then analyzed membrane fatty acid profiles via FAME. The results of this experiment are expressed as the relative percentages of the fatty acids in each sample and are presented in Figure S6D and E, as well as in Table S2. One striking difference across conditions was the difference in the relative proportion of BCFAs to SCFAs (Fig. S6D). After exposure to medium alone (control) for 24 h, BCFAs made up 91.9% of the total fatty acids in the *S. aureus* membrane, while SCFAs accounted for the remaining 8.1% (Fig. S6D). In comparison, treatment with *P. aeruginosa* PA14 wild-type supernatant led to a significantly lower proportion of BCFAs (76.0%), and a correspondingly higher level of SCFAs (24.0%, Fig. S6D).

To determine whether HQNO and siderophores are responsible for the *P. aeruginosa* supernatant-induced shift in *S. aureus* BCFA levels, we examined the lipid composition of *S. aureus* following exposure to supernatant from a mutant that cannot produce HQNO or siderophores. Treatment with supernatant from the *P. aeruginosa* PA14 Δ*pqsL* Δ*pvdA* Δ*pchE* strain (denoted as the ΔΔΔ mutant) led to a BCFA proportion almost identical to that of *S. aureus* exposed to medium alone (91.6% compared to 91.9%, Fig. S6D), indicating that HQNO and siderophores are required for the observed changes in fatty acid levels.

Next, we investigated whether HQNO alone can influence fatty acid composition, and if so, whether it mirrors the effect of *P. aeruginosa* PA14 wild-type supernatant. Indeed, we observed that the addition of HQNO led to levels of BCFAs (74.1%) that were not significantly different from the wild-type supernatant (76.0%, Fig. S6D). These results indicate that exposure to this

small molecule recapitulates the effect of *P. aeruginosa* supernatant on shifting *S. aureus* fatty acid composition.

Furthermore, given the similar effects of HQNO and Antimycin A on increasing *S. aureus* drug sensitivity, we examined whether the addition of Antimycin A led to similar changes in fatty acid composition as compared to treatment with HQNO. The fatty acid profile of Antimycin A-treated cells more closely resembled that of cells exposed to medium alone; the BCFA proportion following treatment with Antimycin A was 90.0%, compared to 91.9% for MEM, and 74.1% for HQNO (Fig. S6D). Thus, unlike HQNO, exposure to Antimycin A did not appear to change *S. aureus* fatty acid composition, thus changes in fatty acid composition did not track with changes in antimicrobial susceptibility.

Similar patterns were observed for the levels of the two BCFA configurations: anteiso and iso. By 24 h, exposure to either medium alone or the mutant supernatant led to similar proportions of anteiso-BCFAs (84.9% and 85.4%, respectively, Fig. S6E). In contrast, *P. aeruginosa* PA14 wild-type supernatant and HQNO-treated samples both had a similarly lower relative proportion of anteiso-BCFA (63.0% and 62.0%, respectively) that was significantly lower compared to growth in MEM (Fig. S6E). As observed above, treatment with Antimycin A mirrored exposure to medium alone; cells exposed to Antimycin A had similar anteiso-BCFA levels as cells grown in medium alone (81.8% and 84.9%, respectively, Fig. S6E). Thus, these data indicate that exposure of *S. aureus* to either *P. aeruginosa* PA14 wild-type supernatant or HQNO decreased levels of anteiso-BCFA relative to the control, but exposure to Antimycin A, which also increases sensitivity to chloroxylenol, did not promote this change in fatty acid profile.

Together, these results indicate that *S. aureus* grown in medium alone exhibits a shift in fatty acid composition after prolonged incubation, and this shift is absent in *P. aeruginosa* PA14 wild-type supernatant or HQNO-exposed *S. aureus* cells.

Supplemental Materials and Methods

Preparation of *P. aeruginosa* supernatants. Overnight liquid cultures of *P. aeruginosa* were diluted to an OD_{600} of 0.05, washed in phosphate-buffered saline (PBS), and resuspended in minimal essential medium (MEM, ThermoFisher Scientific) supplemented with 2 mM L-glutamine (MEM + L-Gln). Plastic 6-well plates were inoculated with 2 ml of the *P. aeruginosa* suspensions per well and incubated at 37°C, 5% CO_2 for 22-24 h at 37°C, 5% CO_2. Afterwards, the culture supernatant were collected, centrifuged at 5,000 x *g* for 5 min, and filter-sterilized through a 0.22-μm filter. In subsequent experiments, wells containing *P. aeruginosa* supernatant received half the well volume of supernatant and half the well volume of either MEM + L-Gln or antibiotic solutions in MEM + L-Gln.

Biolog MicroArray antibiotic susceptibility assay. To test *S. aureus* antibiotic susceptibility versus a range of antimicrobial agents, we utilized the Phenotype MicroArray Panels 11 – 20 from Biolog, which are chemical sensitivity tests for bacteria (panels 1 – 10 are metabolic tests, while panels 21 – 25 are chemical sensitivity tests for fungi). Overnight liquid cultures of *S. aureus* Newman were diluted to an OD_{600} of 0.05, washed in PBS, and resuspended in MEM + L-Gln. For each Phenotype MicroArray panel, half-area 96-well plastic plates were inoculated with 50 μl of *S. aureus* suspension and either 50 μl of MEM + L-Gln or 50 μl of *P. aeruginosa* PA14 wild-type supernatant. The plates were incubated at 37°C, 5% CO_2. The planktonic cell population was collected 24 h post-inoculation (p.i.), serially diluted 10-fold in PBS, and plated on mannitol salt agar. To collect the remaining biofilm cell population from the 96-well plates, 50 μl of 0.1% Triton X-100 in PBS was added to each well. Next, the plates were gently agitated on an undulating rocker for 60 min. Biofilms were further disrupted by covering the plates with a

foil seal and vortexing for 2 min. Biofilm cells were serially diluted and plated as described for the planktonic cells. Agar plates were incubated 37°C for 18 h and planktonic and biofilm CFU were determined. Increased efficacy of a drug was defined as at least a 10-fold decrease in CFU between *S. aureus* exposed to the antibiotic alone and *S. aureus* exposed to *P. aeruginosa* supernatant plus the antibiotic.

Biofilm disruption assay on plastic. *S. aureus* overnight cultures were diluted to an optical density at 600 nm (OD_{600}) of 0.05, and washed once in 1x phosphate-buffered saline (PBS). Bacterial suspensions were prepared in minimal essential medium (MEM; Thermo Fisher Scientific) supplemented with 2 mM L-Glutamine (MEM + L-Gln). Triplicate wells of plastic 96-well plates were inoculated with 100 µl of the bacterial suspension and incubated at 37°C, 5% CO_2 for 6 h. Afterwards, unattached cells were removed and dilutions of antibiotics or compounds at the indicated concentrations in MEM + L-Gln, bacterial supernatants, and MEM + L-Gln were added to the appropriate wells (total well volume of 90 µl). Plates were incubated at 37°C, 5% CO_2 for 18 additional h. Planktonic cells were collected 24 h p.i., serially diluted 10-fold in PBS, and plated on mannitol salt agar. Biofilm cells were collected by adding 50 µl of PBS to each well and mechanical disruption using a solid multipin replicator. Biofilm cells were serially diluted and plated as described for the planktonic cells. Agar plates were incubated 37°C for 18 h and planktonic and biofilm CFU were determined. Viable cell counts for all relevant experiments are reported as log_{10}-transformed CFU per milliliter.

Membrane potential measurements. *S. aureus* membrane potential was determined using the fluorescent dye $DiOC_2$ as previously described (3, 4) with some modifications. Overnight liquid

cultures of *S. aureus* Newman were diluted to an OD_{600} of 0.3, washed in PBS, and resuspended in MEM + L-Gln. Bacterial suspensions were transferred to 6-well plates and treated with MEM + L-Gln alone, CCCP at 5 μM, HQNO at 100 μg/ml, Antimycin A at 100 μg/ml, or *P. aeruginosa* PA14 wild-type or Δ*pqsLpvdApchE* deletion mutant supernatant (total well volume of 2 ml). After incubation at 37°C, 5% CO_2 for 24 h, planktonic *S. aureus* cells were collected (1 ml per sample) and $DiOC_2$ was added to a final concentration of 30 μM. Cells were incubated for 30 min at room temperature and $DiOC_2$ fluorescence was measured at 680 nm following excitation at 485 nm. Additionally, the absorbance at 600 nm was measured. Results are reported as the following ratio: Fluorescence / OD_{600}.

Laurdan membrane fluidity analysis. *S. aureus* membrane fluidity was determined by Laurdan generalized polarization (GP) as previously described (4, 17) with some modifications. Overnight liquid cultures of *S. aureus* Newman were diluted to a final OD_{600} of 1, washed once in PBS, and resuspended in 2 ml of PBS. The 2 ml cell suspension was stained with 10 μM Laurdan for 10 min at room temperature in the dark under agitation. Following staining, cells were washed four times with 2 ml of PBS. Following the final wash, 500 μl of the supernatant was transferred to a separate tube to be used as a blank. The rest of the supernatant was discarded, and the cell pellet was resuspended in 1 ml of PBS. Next, 100 μl of the cell suspension was added to either 100 μl of MEM + L-Gln alone or 100 μl of dilutions of the following compounds in MEM + L-Gln at the indicated final concentrations: benzyl alcohol (at 50 mM, 100 mM, 200 mM, 500 mM, or 1 M), HQNO (at 100 μg/ml, 33 μg/ml, or 11 μg/ml), Antimycin A (at 100 μg/ml), or DMSO and ethanol controls for HQNO and Antimycin A, respectively. Samples or blanks were transferred to replicate wells of clear flat bottomed 96-well

plates (150 µl per well; 3 technical replicates), followed by incubation for 1 h at room temperature in the dark. Laurdan fluorescence was measured at 460 and 500 nm following excitation at 330 nm. Laurdan GP was calculated using the following formula: GP = $(I_{460}-I_{500})/(I_{460}+I_{500})$.

Fatty acid methyl ester analysis. Overnight liquid cultures of *S. aureus* Newman were diluted to an OD_{600} of 0.5, washed in PBS, and resuspended in either MEM + L-Gln, 100 µg/ml HQNO in MEM + L-Gln, 100 µg/ml Antimycin A in MEM + L-Gln, or *P. aeruginosa* PA14 wild-type or Δ*pqsLpvdApchE* deletion mutant supernatant (total volume of 50 ml). Duplicate 250 ml flasks were inoculated with the *S. aureus* suspensions and incubated at 37°C, shaking at 225 rpm. *S. aureus* cells were collected 30 min, 1 h, 3 h, 6 h, 10 h, and 24 h p.i. Cell pellets were washed 2x in cold distilled water, snap-frozen, and stored at -80°C. Whole-cell direct fatty acid methyl ester (FAME) analysis was performed by Microbial ID, Inc. (Newark, DE) as previously described (18). The fatty acids in the bacterial pellets (30-40 mg wet weight) were saponified and methylated, and extracted. Fatty acid methyl esters were then separated using an Agilent 5890 dual-tower gas chromatograph. Fatty acids were identified using the MIDI Sherlock 4.5 microbial identification system.

Literature Cited

1. **Smith L**. 1954. Bacterial cytochromes; difference spectra. Arch Biochem Biophys **50**:299–314.

2. **Lightbown JW**, **Jackson FL**. 1956. Inhibition of cytochrome systems of heart muscle and certain bacteria by the antagonists of dihydrostreptomycin: 2-alkyl-4-hydroxyquinoline N-oxides. Biochem J **63**:130–137.

3. **Nair DR**, **Monteiro JM**, **Memmi G**, **Thanassi J**, **Pucci M**, **Schwartzman J**, **Pinho MG**, **Cheung AL**. 2015. Characterization of a novel small molecule that potentiates □-lactam activity against gram-positive and gram-negative pathogens. Antimicrob Agents Chemother **59**:1876–1885.

4. **Müller A**, **Wenzel M**, **Strahl H**, **Grein F**, **Saaki TNV**, **Kohl B**, **Siersma T**, **Bandow JE**, **Sahl H-G**, **Schneider T**, **Hamoen LW**. 2016. Daptomycin inhibits cell envelope synthesis by interfering with fluid membrane microdomains. Proc Natl Acad Sci USA **113**:E7077–E7086.

5. **Hazan R**, **Que YA**, **Maura D**, **Strobel B**, **Majcherczyk PA**, **Hopper LR**, **Wilbur DJ**, **Hreha TN**, **Barquera B**, **Rahme LG**. 2016. Auto poisoning of the respiratory chain by a quorum-sensing-regulated molecule favors biofilm formation and antibiotic tolerance. Curr Biol **26**:195–206.

6. **Filkins LM**, **Graber JA**, **Olson DG**, **Dolben EL**, **Lynd LR**, **Bhuju S**, **O'Toole GA**. 2015. Coculture of *Staphylococcus aureus* with *Pseudomonas aeruginosa* drives *S. aureus* towards fermentative metabolism and reduced viability in a cystic fibrosis model. J Bacteriol **197**:2252–2264.

7. **White DC**, **Frerman FE**. 1968. Fatty acid composition of the complex lipids of *Staphylococcus aureus* during the formation of the membrane-bound electron transport system. J Bacteriol **95**:2198–2209.

8. **Sen S**, **Sirobhushanam S**, **Johnson SR**, **Song Y**, **Tefft R**, **Gatto C**, **Wilkinson BJ**. 2016. Growth-environment dependent modulation of *Staphylococcus aureus* branched-chain to straight-chain fatty acid ratio and incorporation of unsaturated fatty acids. PLoS ONE **11**:e0165300.

9. **Keweloh H**, **Diefenbach R**, **Rehm H-JR**. 1991. Increase of phenol tolerance of *Escherichia coli* by alterations of the fatty acid composition of the membrane lipids. Arch Microbiol **157**:49–53.

10. **Willecke K**, **Pardee AB**. 1971. Fatty acid-requiring mutant of *Bacillus subtilis* defective in branched chain alpha-keto acid dehydrogenase. J Biol Chem **246**:5264–5272.

11. **Legendre S**, **Letellier L**, **Shechter E**. 1980. Influence of lipids with branched-chain fatty acids on the physical, morphological and functional properties of *Escherichia coli*

cytoplasmic membrane. Biochimica et Biophysica Acta (BBA) - Biomembranes **602**:491–505.

12. **Kaneda T**. 1991. Iso- and anteiso-fatty acids in bacteria: biosynthesis, function, and taxonomic significance. Microbiol Rev **55**:288–302.

13. **Singh VK**, **Hattangady DS**, **Giotis ES**, **Singh AK**, **Chamberlain NR**, **Stuart MK**, **Wilkinson BJ**. 2008. Insertional inactivation of branched-chain-keto acid dehydrogenase in *Staphylococcus aureus* leads to decreased branched-chain membrane fatty acid content and increased susceptibility to certain stresses. Appl Environ Microbiol **74**:5882–5890.

14. **Kaiser JC**, **Sen S**, **Sinha A**, **Wilkinson BJ**, **Heinrichs DE**. 2016. The role of two branched-chain amino acid transporters in *Staphylococcus aureus* growth, membrane fatty acid composition and virulence. Mol Microbiol **102**:850–864.

15. **Lobasso S**, **Palese LL**, **Angelini R**, **Corcelli A**. 2013. Relationship between cardiolipin metabolism and oxygen availability in *Bacillus subtilis*. FEBS Open Bio **3**:151–155.

16. **Unsay JD**, **Cosentino K**, **Subburaj Y**, **García-Sáez AJ**. 2013. Cardiolipin effects on membrane structure and dynamics. Langmuir **29**:15878–15887.

17. **Strahl H**, **Bürmann F**, **Hamoen LW**. 2014. The actin homologue MreB organizes the bacterial cell membrane. Nat Commun **5**:3442.

18. **Zhu K**. 2005. Precursor and temperature modulation of fatty acid composition and growth of *Listeria monocytogenes* cold-sensitive mutants with transposon-interrupted branched-chain-keto acid dehydrogenase. Microbiology **151**:615–623.
