# Supplementary material for: Pseudomonas aeruginosa Increases the Sensitivity of Biofilm-Grown Staphylococcus aureus to Membrane-Targeting Antiseptics and Antibiotics
Source: mBio. 2019 Jul 30;10(4):e01501-19. doi: 10.1128/mBio.01501-19 (PMC6667622; doi:10.1128/mBio.01501-19)
Supplement: FIG S1 [file mBio.01501-19-sf001.pdf]

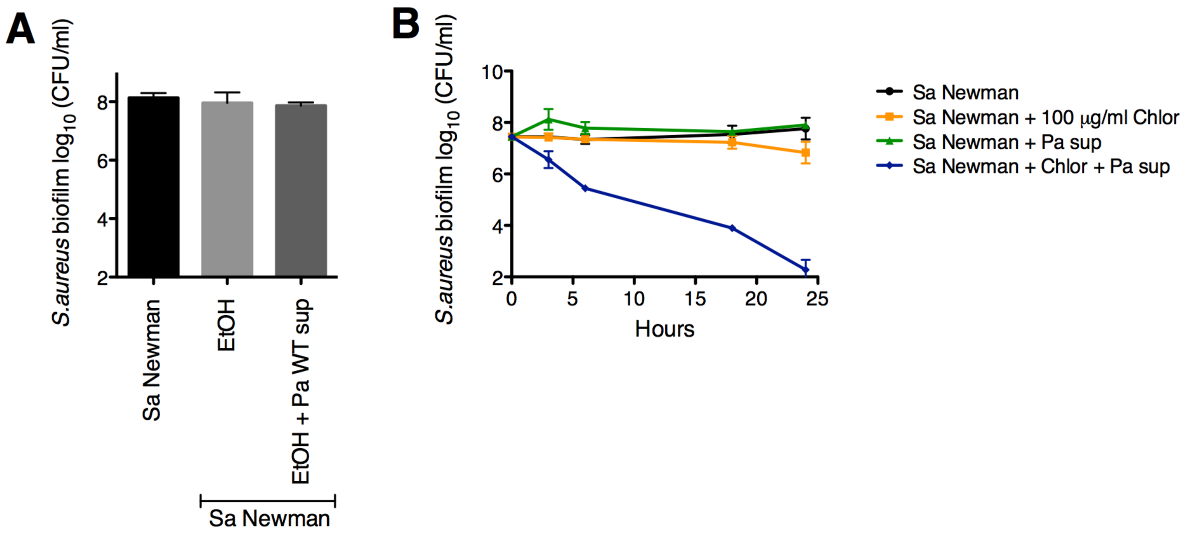

Figure S1. The concentration of ethanol used in this study does not decrease *S. aureus* biofilm viability in the presence or absence of *P. aeruginosa* supernatant and *P. aeruginosa* supernatant rapidly increases *S. aureus* biofilm sensitivity to chloroxylenol. (**A**) Biofilm disruption assays on plastic were performed with *S. aureus* (Sa) Newman, *P. aeruginosa* PA14 supernatant (Pa sup), and ethanol control for chloroxylenol at 100 µg/ml (ethanol is used to dissolve chloroxylenol). Biofilms were grown for 6 hours, exposed to the above treatments for 18 hours, and *S. aureus* biofilm CFU were determined. Each column displays the average from four biological replicates, each with three technical replicates. Error bars indicate SD. (**B**) Biofilm disruption assays on plastic were performed with *S. aureus* (Sa) Newman, *P. aeruginosa* PA14 supernatant (Pa sup), and chloroxylenol (Chlor) at 100 µg/ml under normoxic or anoxic conditions. Biofilms were grown for 6 hours, exposed to the above treatments for 0, 3, 6, 18, or 24 hours, and *S. aureus* biofilm CFU were determined. Each time point displays the average from at least two biological replicates, each with three technical replicates. Error bars indicate standard deviation (SD).
