# Supplementary material for: Pseudomonas aeruginosa Increases the Sensitivity of Biofilm-Grown Staphylococcus aureus to Membrane-Targeting Antiseptics and Antibiotics
Source: mBio. 2019 Jul 30;10(4):e01501-19. doi: 10.1128/mBio.01501-19 (PMC6667622; doi:10.1128/mBio.01501-19)
Supplement: TABLE S2 [file mBio.01501-19-st002.pdf]

Table S2. Strains used in this study.

Species and strain	Strain number	Phenotype	Source / reference
S. aureus			
Newman	SMC 1007	MSSA[a]	(1)
JE2	Sa TML[c]	MRSA[b]	(2)
JE2 NE592 (*atpA*::tn)	Sa TML		(2)
JE2 NE1548 (*atpB*::tn)	Sa TML		(2)
JE2 NE1850 (*atpG*::tn)	Sa TML		(2)
JE2 NE1889 (*atpH*::tn)	Sa TML		(2)
JE2 NE12* (*lmrS*::tn)	Sa TML		(2)
JE2 NE17*	Sa TML		(2)
JE2 NE179* (*mdeA*::tn)	Sa TML		(2)
JE2 NE370*	Sa TML		(2)
JE2 NE405* (*mepA*::tn)	Sa TML		(2)
JE2 NE531* (*sdrM*::tn)	Sa TML		(2)
JE2 NE749* (*sbnD*::tn)	Sa TML		(2)
JE2 NE773*	Sa TML		(2)
JE2 NE781*	Sa TML		(2)
JE2 NE901*	Sa TML		(2)
JE2 NE1034* (*norA*::tn)	Sa TML		(2)
JE2 NE1041*	Sa TML		(2)
JE2 NE1184* (*norB*::tn)	Sa TML		(2)
JE2 NE1400*	Sa TML		(2)
JE2 NE1804* (*norC*::tn)	Sa TML		(2)
JE2 NE1616* (*sepA*::tn)	Sa TML		(2)
JE2 NE1287 (*cls*::tn)	Sa TML		(2)
JE2 NE258 (*cls*::tn)	Sa TML		(2)
Col	SMC 6598	MRSA	(3)
Col *hemB* mutant	SMC 6599, ALC 4689	MRSA, SCV[d]	(4)
SH1000		MSSA	(5)
SH1000 *lpd*::Kan			(5)
USA300		MRSA	(6)
USA300 Δ*brnQ1*			(6)
USA300 Δ*brnQ2*			(6)
USA300 Δ*brnQ1* Δ*brnQ2* *brnQ3*::Tet			(6)
Clinical isolate	SMC 1597	MSSA	(7)
Clinical isolate	SMC 1603	MSSA	(7)
Clinical isolate	SMC 1605	MSSA	(7)
Clinical isolate	SMC 6531	MRSA	A. L. Cheung
Clinical isolate	SMC 6532	MRSA	A. L. Cheung
Clinical isolate	SMC 6533	MRSA	A. L. Cheung

P. aeruginosa			
PA14	SMC 232	non-mucoid	(8)
PA14 Δ*pqsA*	SMC 5013		L. G. Rahme
PA14 Δ*pqsH*	SMC 5017		(9)
PA14 Δ*pqsL*	SMC 6216		(7)
PA14 Δ*pvdA*	SMC 6596		(7)
PA14 Δ*pchE*	SMC 6597		(7)
PA14 Δ*pvdA*Δ*pchE*	SMC 6215		(10)
PA14 Δ*pqsL*Δ*pvdA*Δ*pchE*	SMC 6219		(7)
PA14 *hcnA*::tn	Pa TML[e]		(11)
PA14 *hcnB*::tn	Pa TML		(11)
PA14 *lasA*::tn	Pa TML		(11)
PA14 *lasB*::tn	Pa TML		(11)
PA14 *rhlA*::tn	Pa TML		(11)

[a] MSSA, methicillin-sensitive *S. aureus*
[b] MRSA, methicillin-resistant *S. aureus*
[c] Sa TML, *S. aureus* JE2 Nebraska transposon mutant library
[d] SCV, small colony variant
[e] Pa TML, *P. aeruginosa* PA14 NR transposon mutant library
* *S. aureus* antibiotic transporter mutants

References

1. **Duthie ES**. 1952. Variation in the antigenic composition of staphylococcal coagulase. J Gen Microbiol **7**:320–326.

2. **Fey PD**, **Endres JL**, **Yajjala VK**, **Widhelm TJ**, **Boissy RJ**, **Bose JL**, **Bayles KW**. 2013. A genetic resource for rapid and comprehensive phenotype screening of nonessential *Staphylococcus aureus* genes. MBio **4**:e00537–12.

3. **Dyke KG**, **Jevons MP**, **Parker MT**. 1966. Penicillinase production and intrinsic resistance to penicillins in *Staphylococcus aureus*. Lancet **1**:835–838.

4. **Vaudaux P**, **Francois P**, **Bisognano C**, **Kelley WL**, **Lew DP**, **Schrenzel J**, **Proctor RA**, **McNamara PJ**, **Peters G**, **Eiff von C**. 2002. Increased expression of clumping factor and fibronectin-binding proteins by *hemB* mutants of *Staphylococcus aureus* expressing small colony variant phenotypes. Infect Immun **70**:5428–5437.

5. **Singh VK**, **Hattangady DS**, **Giotis ES**, **Singh AK**, **Chamberlain NR**, **Stuart MK**, **Wilkinson BJ**. 2008. Insertional inactivation of branched-chain-keto acid dehydrogenase in *Staphylococcus aureus* leads to decreased branched-chain membrane fatty acid content and increased susceptibility to certain stresses. Appl Environ Microbiol **74**:5882–5890.

6. **Kaiser JC**, **Omer S**, **Sheldon JR**, **Welch I**, **Heinrichs DE**. 2015. Role of BrnQ1 and BrnQ2 in branched-chain amino acid transport and virulence in *Staphylococcus aureus*. Infect Immun **83**:1019–1029.

7. **Filkins LM**, **Graber JA**, **Olson DG**, **Dolben EL**, **Lynd LR**, **Bhuju S**, **O'Toole GA**. 2015. Coculture of *Staphylococcus aureus* with *Pseudomonas aeruginosa* drives *S. aureus* towards fermentative metabolism and reduced viability in a cystic fibrosis model. J Bacteriol **197**:2252–2264.

8. **Rahme LG**, **Stevens EJ**, **Wolfort SF**, **Shao J**, **Tompkins RG**, **Ausubel FM**. 1995. Common virulence factors for bacterial pathogenicity in plants and animals. Science **268**:1899–1902.

9. **Cugini C**, **Morales DK**, **Hogan DA**. 2010. *Candida albicans*-produced farnesol stimulates *Pseudomonas* quinolone signal production in LasR-defective *Pseudomonas aeruginosa* strains. Microbiology (Reading, Engl) **156**:3096–3107.

10. **Wang Y**, **Wilks JC**, **Danhorn T**, **Ramos I**, **Croal L**, **Newman DK**. 2011. Phenazine-1-carboxylic acid promotes bacterial biofilm development via ferrous iron acquisition. J Bacteriol **193**:3606–3617.

11. **Liberati NT**, **Urbach JM**, **Miyata S**, **Lee DG**, **Drenkard E**, **Wu G**, **Villanueva J**, **Wei T**, **Ausubel FM**. 2006. An ordered, nonredundant library of *Pseudomonas aeruginosa* strain PA14 transposon insertion mutants. Proceedings of the National Academy of Sciences **103**:2833–2838.
