# Supplementary material for: Pseudomonas aeruginosa Increases the Sensitivity of Biofilm-Grown Staphylococcus aureus to Membrane-Targeting Antiseptics and Antibiotics
Source: mBio. 2019 Jul 30;10(4):e01501-19. doi: 10.1128/mBio.01501-19 (PMC6667622; doi:10.1128/mBio.01501-19)
Supplement: FIG S2 [file mBio.01501-19-sf002.pdf]

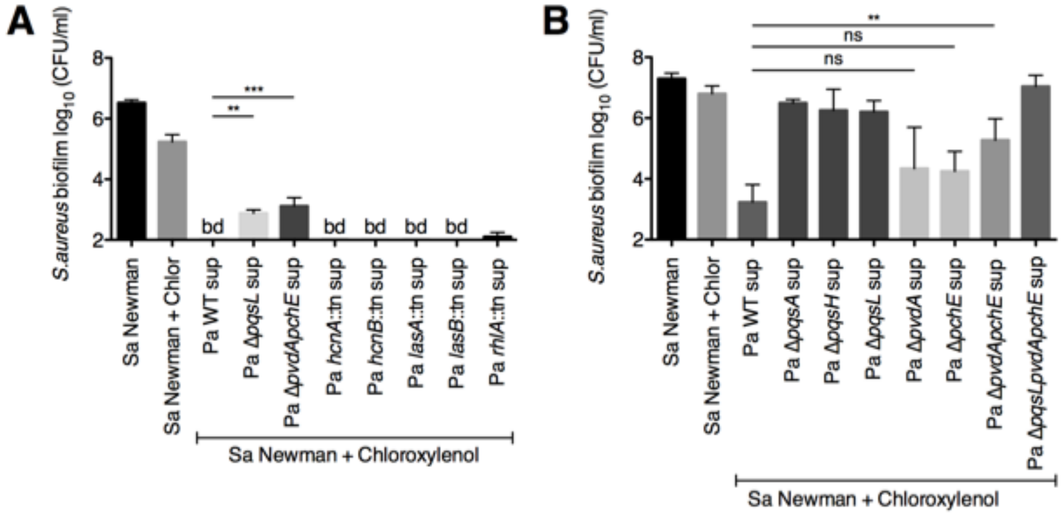

Figure S2. Testing the ability of *P. aeruginosa* PA14 mutants defective in exoproduct production to increase *S. aureus* biofilm sensitivity to chloroxylenol. (**A and B**) Biofilm disruption assays on plastic were performed with *S. aureus* (Sa) Newman, supernatants from *P. aeruginosa* PA14 wild-type and the specified mutants (Pa sup), and chloroxylenol (Chlor) at 100 µg/ml. Biofilms were grown for 6 hours, exposed to the above treatments for 18 hours, and *S. aureus* biofilm CFU were determined. Each column displays the average from at least three biological replicates, each with three technical replicates. Error bars indicate SD. bd, below detection. ns, not significant; **, $P < 0.01$, ***, $P < 0.001$, by ordinary one-way ANOVA and Tukey's multiple comparison post-test.
