# Supplementary material for: Pseudomonas aeruginosa Increases the Sensitivity of Biofilm-Grown Staphylococcus aureus to Membrane-Targeting Antiseptics and Antibiotics
Source: mBio. 2019 Jul 30;10(4):e01501-19. doi: 10.1128/mBio.01501-19 (PMC6667622; doi:10.1128/mBio.01501-19)
Supplement: FIG S3 [file mBio.01501-19-sf003.pdf]

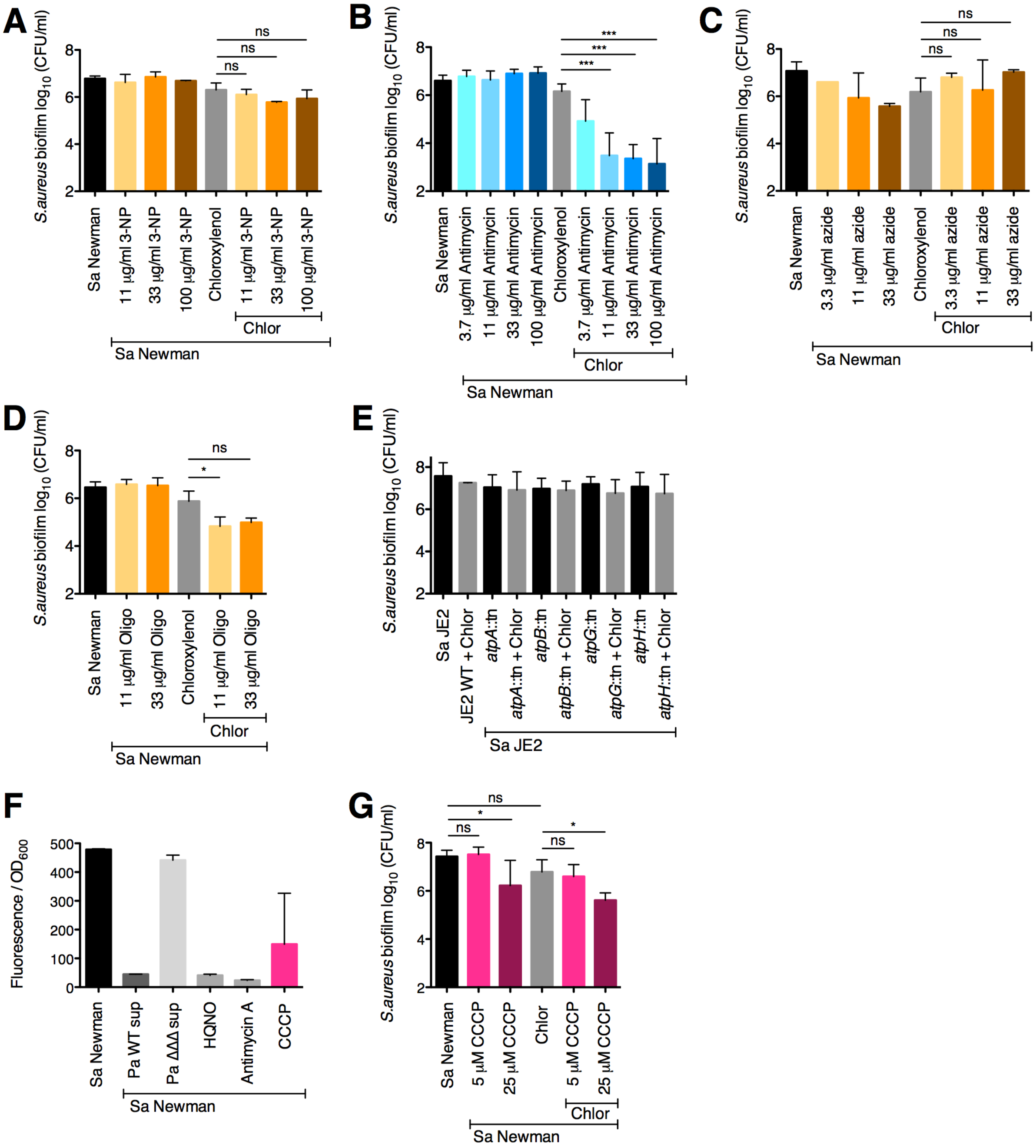

Figure S3. Testing the ability of electron transport chain inhibitors and a proton ionophore to increase *S. aureus* biofilm sensitivity to chloroxylenol. (**A to D**) Biofilm disruption assays on plastic were performed with *S. aureus* (Sa) Newman, chloroxylenol (Chlor) at 100 µg/ml, and the specified concentrations of 3-nitropropionic acid (3-NP) (**A**), Antimycin A (**B**), Sodium azide

(azide) (**C**), or oligomycin (Oligo) (**D**). Biofilms were grown for 6 hours, exposed to the above treatments for 18 hours, and *S. aureus* biofilm CFU were determined. (**E**) Biofilm disruption assays on plastic were performed with *S. aureus* (Sa) JE2 parental strain or the specified transposon mutant, and chloroxylenol (Chlor) at 100 µg/ml. Each column displays the average from at least two biological replicates, each with three technical replicates. Error bars indicate SD. ns, not significant; *, $P < 0.05$, ***, $P < 0.001$, by ordinary one-way ANOVA and Tukey's multiple comparison post-test. (**F**) Membrane potential was measured using the fluorescent dye $DiOC_2$ following exposure to carbonyl cyanide 3-chlorophenylhydrazone (CCCP) at 5 µM, HQNO at 100 µg/ml, Antimycin A at 100 µg/ml, or *P. aeruginosa* PA14 wild-type or Δ*pqsLpvdApchE* deletion mutant supernatant for 24 h. $DiOC_2$ fluorescence was measured at 680 nm following excitation at 485 nm. Results are reported as Fluorescence / OD_{600}. Each column displays the average from two biological replicates, each with three technical replicates. (**G**) Biofilm disruption assays on plastic were performed with *S. aureus* (Sa) Newman, chloroxylenol (Chlor) at 100 µg/ml, and CCCP at 5 µM or 25 µM. Biofilms were grown for 6 hours, exposed to the above treatments for 18 hours, and *S. aureus* biofilm CFU were determined. Each column displays the average from at least two biological replicates, each with three technical replicates. Error bars indicate SD. ns, not significant; *, $P < 0.05$, by ordinary one-way ANOVA and Tukey's multiple comparison post-test.
