# Supplementary material for: Pseudomonas aeruginosa Increases the Sensitivity of Biofilm-Grown Staphylococcus aureus to Membrane-Targeting Antiseptics and Antibiotics
Source: mBio. 2019 Jul 30;10(4):e01501-19. doi: 10.1128/mBio.01501-19 (PMC6667622; doi:10.1128/mBio.01501-19)
Supplement: FIG S4 [file mBio.01501-19-sf004.pdf]

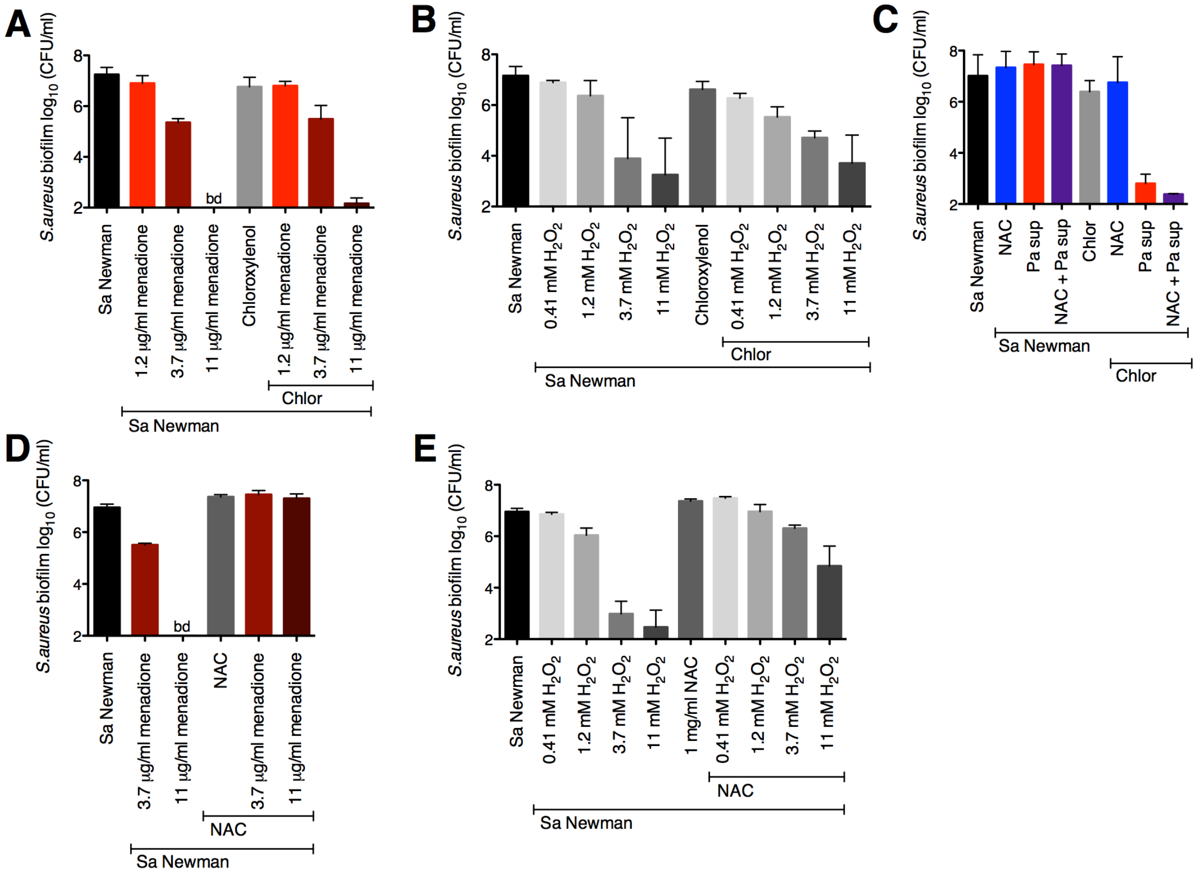

Figure S4. Reactive oxygen species do not sensitize *S. aureus* biofilms to chloroxylenol. (**A to E**) Biofilm disruption assays on plastic were performed with *S. aureus* (Sa) Newman, chloroxylenol (Chlor) at 100 µg/ml, and the specified concentrations of menadione (**A and D**) and hydrogen peroxide (**B and E**), N-acetyl cysteine (NAC) at 1 mg/ml (**C to E**), and *P. aeruginosa* PA14 supernatant (Pa sup) (**C**). Biofilms were grown for 6 hours, exposed to the above treatments for 18 hours, and *S. aureus* biofilm CFU were determined. Each column displays the average from at least two biological replicates, each with three technical replicates. Error bars indicate SD. bd, below detection.
