# Supplementary material for: Pseudomonas aeruginosa Increases the Sensitivity of Biofilm-Grown Staphylococcus aureus to Membrane-Targeting Antiseptics and Antibiotics
Source: mBio. 2019 Jul 30;10(4):e01501-19. doi: 10.1128/mBio.01501-19 (PMC6667622; doi:10.1128/mBio.01501-19)
Supplement: FIG S5 [file mBio.01501-19-sf005.pdf]

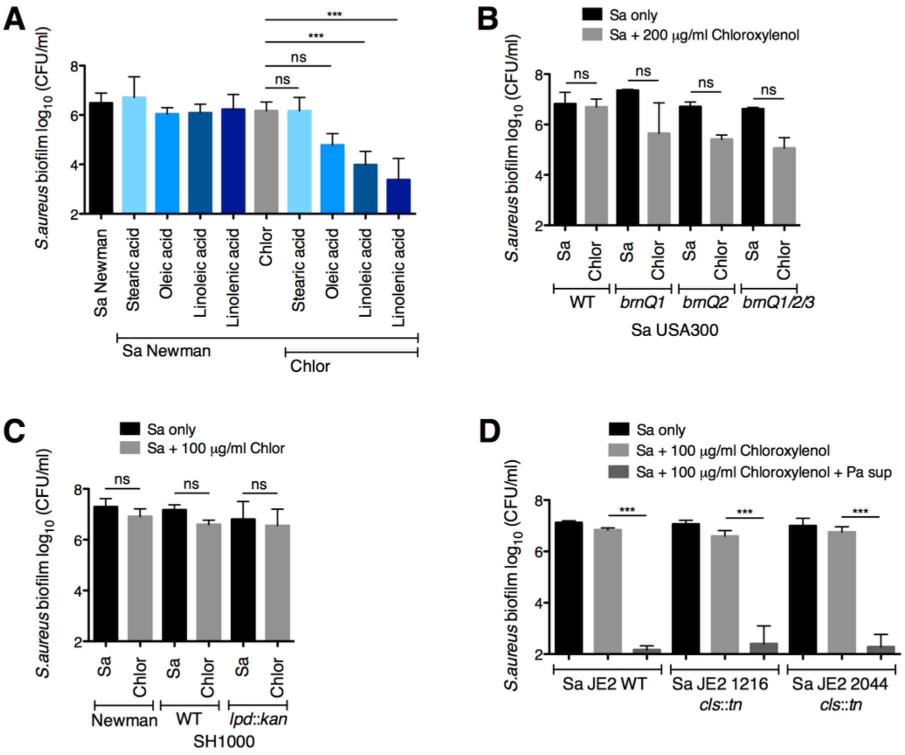

Figure S5. Manipulating membrane fatty acid composition alters *S. aureus* biofilm sensitivity to chloroxylenol. (**A**) Biofilm disruption assays on plastic were performed with *S. aureus* (Sa) Newman, chloroxylenol (Chlor) at 100 μg/ml, and the specified fatty acids at 100 μg/ml. Biofilms were grown for 6 hours, exposed to the above treatments for 18 hours, and *S. aureus* biofilm CFU were determined. (**B to D**) Biofilm disruption assays on plastic were performed with the specified *S. aureus* (Sa) strains and the specified concentrations of chloroxylenol (Chlor). Biofilms were grown for 6 hours, exposed to the above treatments for 18 hours, and *S. aureus* biofilm CFU were determined. Each column displays the average from at least two biological replicates, each with three technical replicates. Error bars indicate SD. ns, not significant; ***, $P < 0.001$, by ordinary one-way ANOVA and Tukey's multiple comparison post-test.
