# Supplementary material for: Pseudomonas aeruginosa Increases the Sensitivity of Biofilm-Grown Staphylococcus aureus to Membrane-Targeting Antiseptics and Antibiotics
Source: mBio. 2019 Jul 30;10(4):e01501-19. doi: 10.1128/mBio.01501-19 (PMC6667622; doi:10.1128/mBio.01501-19)
Supplement: FIG S6 [file mBio.01501-19-sf006.pdf]

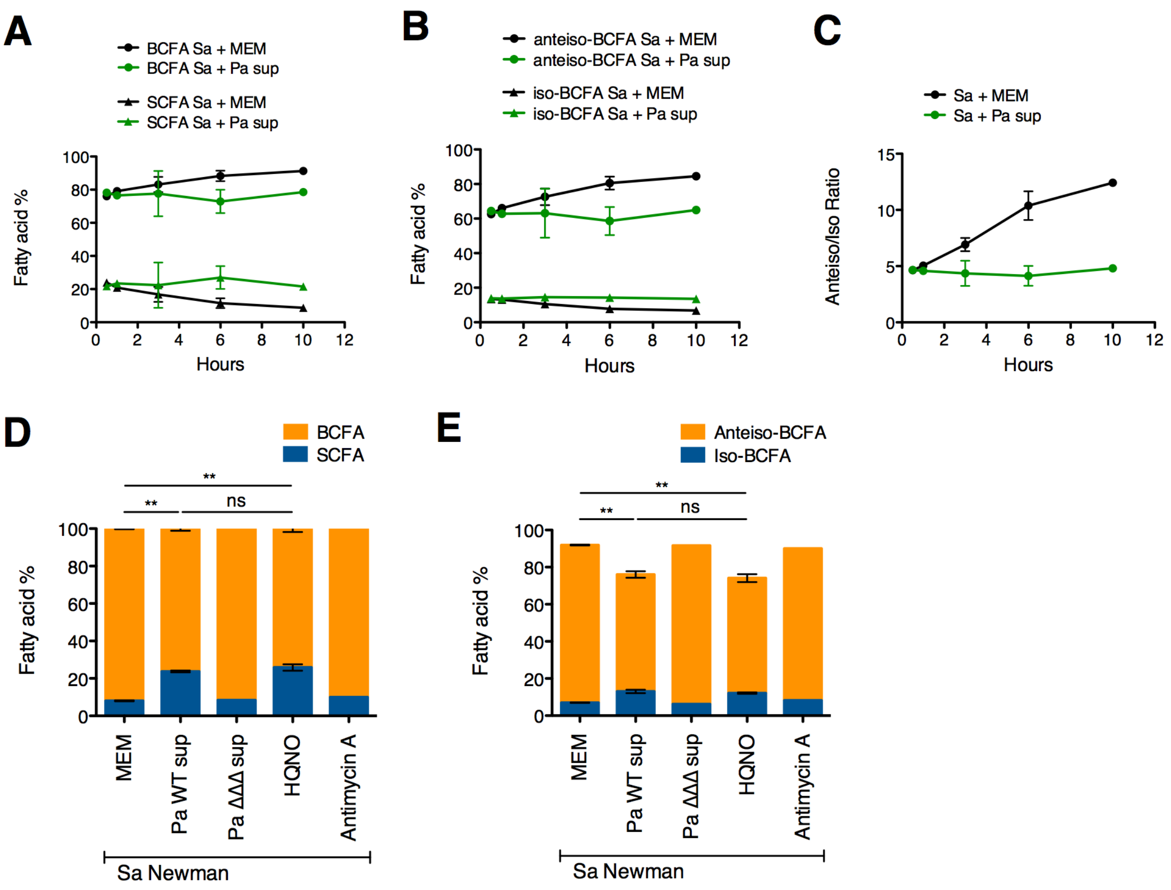

Figure S6. Exposure to *P. aeruginosa* exoproducts alters *S. aureus* membrane fatty acid composition. (**A to C**) *S. aureus* fatty acid composition was determined over time by performing fatty acid methyl ester (FAME) analysis following exposure of *S. aureus* (Sa) Newman to either medium alone (MEM) or *P. aeruginosa* PA14 supernatant (Pa sup) for the indicated amount of time. The relative percentages of branched-chain fatty acids (BCFA) and straight-chain fatty acids (SCFA) were reported (**A**). The relative percentages of anteiso- and iso-BCFA (**B**) and the ratio of these fatty acids (**C**) are also shown. Data in panels A, B, and C were from the same experiments. The 3 h and 6 h time points display the average from two biological replicates, while the 30 min, 1 h, and 10 h time points display a single biological replicate. Error bars indicate standard deviation (SD). At the 3 h and 6 h time points, BCFA averages were compared

between samples in A, and anteiso-BCFA averages were compared between samples in B; no significant differences in average levels of BCFA or anteiso-BCFA were found between *S. aureus* Newman exposed to *P. aeruginosa* supernatant or medium alone by two-tailed unpaired t test. (**D and E**) *S. aureus* fatty acid composition was measured via FAME analysis following exposure of *S. aureus* (Sa) Newman to either medium alone (MEM), supernatants from wild-type *P. aeruginosa* PA14 supernatant (Pa WT sup) or the Δ*pqsL* Δ*pvdA* Δ*pchE* mutant (Pa ΔΔΔ sup), HQNO at 100 μg/ml, or Antimycin A at 100 μg/ml for 24 h. Shown are the relative percentages of branched-chain fatty acids (BCFA) vs. straight-chain fatty acids (SCFA) (**D**), and the relative percentages of anteiso- vs. iso-BCFA (**E**). Data in panels D and E were from the same experiments. The MEM, Pa WT sup, and HQNO columns display the average from two biological replicates, each with two technical replicates, while the Pa ΔΔΔ sup and Antimycin A columns display a single biological replicate, each with two technical replicates. Error bars indicate standard deviation (SD). BCFA averages were compared between samples in D, and anteiso-BCFA averages were compared between samples in E; **, $P < 0.01$, by ordinary one-way ANOVA and Tukey's multiple comparison post-test.
